# Imaging side effects and complications of chemotherapy and radiation therapy: a pictorial review from head to toe

**DOI:** 10.1186/s13244-021-01017-2

**Published:** 2021-06-10

**Authors:** Domenico Albano, Massimo Benenati, Antonio Bruno, Federico Bruno, Marco Calandri, Damiano Caruso, Diletta Cozzi, Riccardo De Robertis, Francesco Gentili, Irene Grazzini, Giuseppe Micci, Anna Palmisano, Carlotta Pessina, Paola Scalise, Federica Vernuccio, Antonio Barile, Vittorio Miele, Roberto Grassi, Carmelo Messina, Young SIRM Working Group, Domenico Albano, Massimo Benenati, Antonio Bruno, Federico Bruno, Marco Calandri, Damiano Caruso, Diletta Cozzi, Riccardo De Robertis, Francesco Gentili, Irene Grazzini, Giuseppe Micci, Anna Palmisano, Carlotta Pessina, Paola Scalise, Federica Vernuccio, Carmelo Messina

**Affiliations:** 1 IRCCS Istituto Ortopedico Galeazzi, Via Riccardo Galeazzi 4, 20161 Milan, Italy; 2 Sezione di Scienze Radiologiche, Dipartimento di Biomedicina, Neuroscienze e Diagnostica Avanzata, Università Degli Studi di Palermo, Via del Vespro 127, 90127 Palermo, Italy; 3 Italian Society of Medical and Interventional Radiology (SIRM), SIRM Foundation, Via della Signora 2, 20122 Milan, Italy; 4 Dipartimento di Diagnostica per Immagini, Radioterapia, Oncologia ed Ematologia, Fondazione Policlinico Universitario A. Gemelli IRCCS, Rome, Italy; 5 Diagnostic and Interventional Radiology Unit, Maggiore Hospital “C. A. Pizzardi”, 40133 Bologna, Italy; 6 Department of Biotechnology and Applied Clinical Sciences, University of L’Aquila, 67100 L’Aquila, Italy; 7 Radiology Unit, A.O.U. San Luigi Gonzaga di Orbassano, Department of Oncology, University of Torino, 10043 Turin, Italy; 8 Department of Surgical and Medical Sciences and Translational Medicine, Sapienza University of Rome - Sant’Andrea University Hospital, Via di Grottarossa, 1035-1039, 00189 Rome, Italy; 9 Department of Emergency Radiology, University Hospital Careggi, Largo Brambilla 3, 50123 Florence, Italy; 10 U.O.C. Radiologia BT, Ospedale Civile Maggiore – Azienda Ospedaliera Universitaria Integrata Verona, Piazzale A. Stefani 1, 37126 Verona, Italy; 11 Unit of Diagnostic Imaging, Department of Radiological Sciences, University of Siena, Azienda Ospedaliero-Universitaria Senese, Siena, Italy; 12 Department of Radiology, Section of Neuroradiology, San Donato Hospital, Arezzo, Italy; 13 Experimental Imaging Centre, Radiology Unit, IRCCS San Raffaele Scientific Institute, Milan, Italy; 14 School of Medicine, Vita-Salute San Raffaele University, via Olgettina 58, 20132 Milan, Italy; 15 Department of Radiology, University of Brescia, Piazzale Spedali Civili 1, 25123 Brescia, Italy; 16 Department of Diagnostic Imaging, Pisa University Hospital, Via Paradisa 2, 56124 Pisa, Italy; 17 Department of Precision Medicine, University of Campania “L. Vanvitelli”, 80138 Naples, Italy

**Keywords:** Chemotherapy, Radiotherapy, Magnetic resonance imaging, Complications, Side effects

## Abstract

Newer biologic drugs and immunomodulatory agents, as well as more tolerated and effective radiation therapy schemes, have reduced treatment toxicity in oncology patients. However, although imaging assessment of tumor response is adapting to atypical responses like tumor flare, expected changes and complications of chemo/radiotherapy are still routinely encountered in post-treatment imaging examinations. Radiologists must be aware of old and newer therapeutic options and related side effects or complications to avoid a misinterpretation of imaging findings. Further, advancements in oncology research have increased life expectancy of patients as well as the frequency of long-term therapy-related side effects that once could not be observed. This pictorial will help radiologists tasked to detect therapy-related complications and to differentiate expected changes of normal tissues from tumor relapse.

## Key points

- Oncology treatments induce local and systemic expected changes on normal tissues.
- Several complications may be observed in follow-up imaging examinations of oncology patients.
- Radiologists are tasked to differentiate expected findings from residual/relapse of tumors.

## Background

Daily practice and research in oncology are shifting toward precision medicine, with treatments targeted to specific patient and tumor profiles [1, 2]. This underlying concept of health care enables to personalize chemotherapy (ChT) and radiotherapy (RT) schemes improving safety and effectiveness [3]. Meanwhile, advancements in oncology imaging walk hand-in-hand with new treatment strategies. Fast and highly performing CT and MRI technologies have opened new frontiers in oncology imaging, allowing tissue characterization, early diagnosis, prognostic evaluation, and accurate response assessment; this innovative approach results in shifting the radiological assessment from the mere morphologic evaluation to obtaining qualitative and functional data that can be combined with patients clinical information [4–7].

Nevertheless, oncology treatment regimens may lead to local and systemic changes and complications depending on the type of treatment [8–10]. In this scenario, the correct interpretation of post-treatment imaging exams is crucial for an adequate and prompt management of cancer patients. Indeed, expected changes of normal tissues with specific imaging features are associated with several treatments. This review describes the main imaging changes induced by different treatment strategies in a comprehensive assessment “from head to toe.” The primary aim of this article is to guide radiologists tasked to face with old and new oncological therapies in the interpretation of post-treatment imaging exams for an adequate differentiation of expected post-treatment changes from pathological findings.

## Imaging findings

### Central nervous system (CNS)

CNS toxicity of anticancer treatments has received growing interest over the last years, as severe neurotoxicity may affect up to 33% of patients [11]. It may occur after partial or whole-brain RT and ChT, due to a direct or an indirect injury (e.g., coagulopathy). MRI is the modality of choice to differentiate therapy-related side effects from disease progression, infections, and paraneoplastic syndromes [12].

#### RT-induced neurotoxicity

**Fig. 1:**
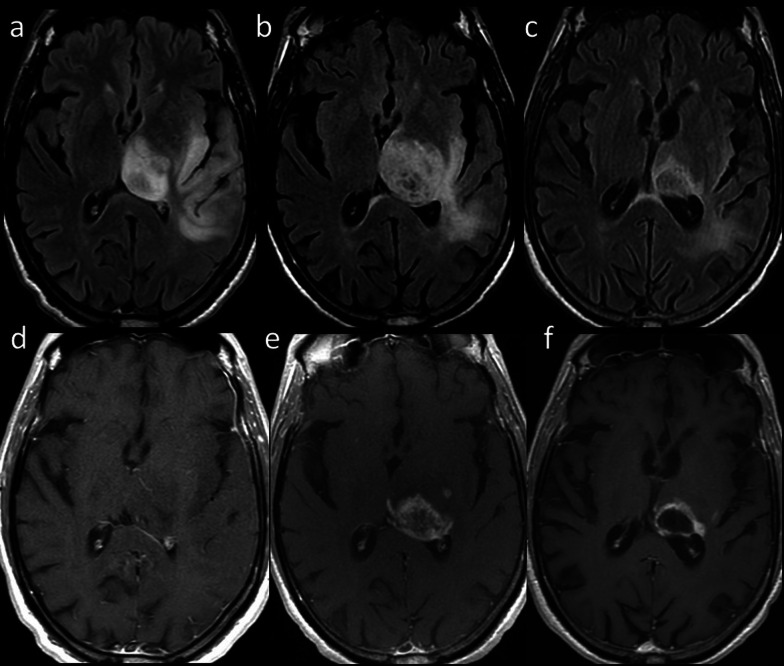
Pseudoprogression. 71-year-old man with glioblastoma treated with ChT and RT. Axial FLAIR (upper row) and contrast-enhanced T1-weighted (lower row) images before (**a**, **d**), during (**b**, **e**), and 3 months after treatment (**c**, **f**) showed initial occurrence of increasing size and enhancement of the lesion, followed by reduction of both signal changes and enhancement at the 3-month follow-up

**Fig. 2:**
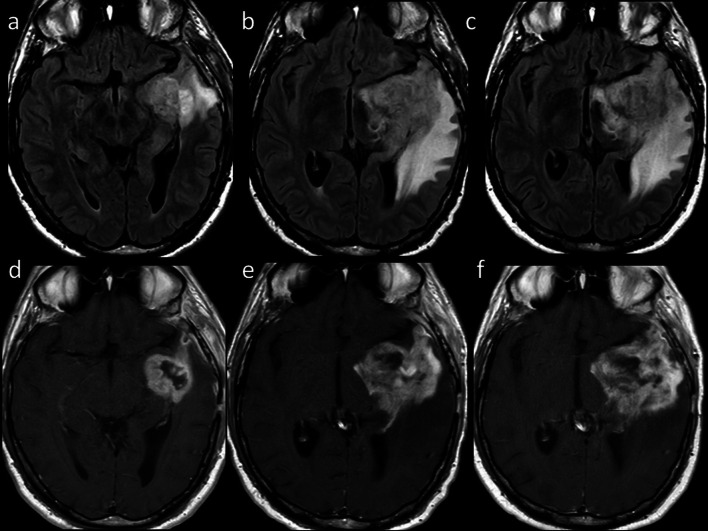
True progression. 53-year-old man with glioblastoma treated with surgery, ChT and RT. Axial FLAIR (upper row) and contrast-enhanced T1-weighted (lower row) 1 month after surgery (**a**, **d**), and 1 (**b**, **e**) and 4 (**c**, **f**) months after ChT/RT showed continuous increasing in signal changes and enhancement of the lesion

RT-induced brain injury can be divided into acute, early delayed, and late-delayed (Table 1) [11, 13]. Acute brain injury is rare with conventional dose fractionation schemes and no changes are generally observed on MRI [11, 14]. In early delayed brain injury, T2-hyperintense areas and new abnormal enhancement patterns may be detected, a phenomenon known as pseudoprogression and classically described in high-grade gliomas after initiation of treatment with RT and ChT, most commonly temozolomide [13]. Advanced MRI techniques allow to differentiate true progression from these transient post-treatment changes, with the latter showing higher ADC signal and lower rCBV on perfusion compared with viable tumor, as well as a decrease in total tumor burden during follow-up (Figs. 1, 2) [14].

**Table 1:** Timeline, symptoms, and imaging findings of radiation-induced brain injury [11–14]

	Acute	Early Delayed	Late delayed
Onset	Days/weeks after RT Reversible	1–4 months after RT Reversible	6 months after RT Not reversible
Symptoms	Headache Nausea and vomiting Transient neurological deficits	Somnolence Transient cognitive deficits and memory loss	Focal or diffuse neurological defects Progressive cognitive impairment
Imaging findings	Inapparent	Inapparent Pseudoprogression	Leukoencephalopathy Parenchymal atrophy Radionecrosis Vascular disorders (stroke, Moyamoya, mineralizing microangiopathy, capillary telangiectasias, cavernous malformations) Secondary tumors Transient focal enhancing lesions Stroke-like migraine attacks after radiation therapy (SMART)

According to the imaging pattern, late-delayed reactions can be classified as follows.

**Fig. 3:**
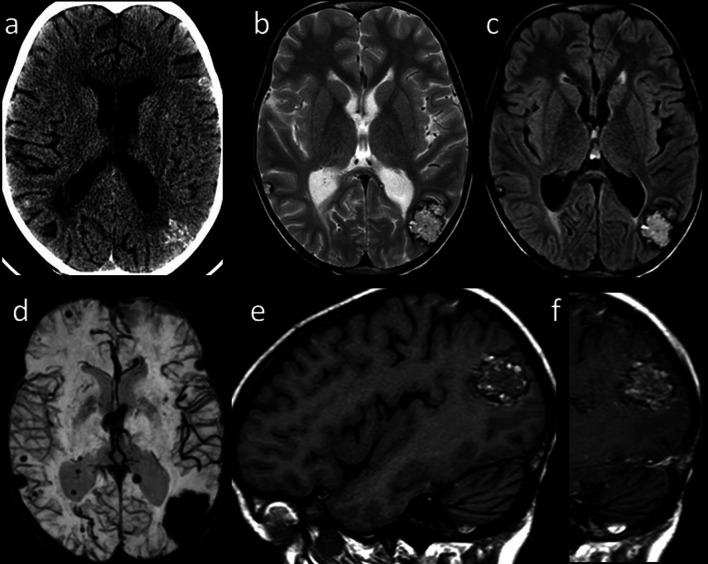
Radiation-induced vascular malformations (1). Axial CT image (**a**) of a 15-year-old male, treated for medulloblastoma 10 years before with surgery, whole-brain RT and ChT, shows multiple hyperdense brain lesions. Axial T2-weighted (**b**) and FLAIR (**c**) images show corresponding inhomogeneous lesions, and SWI minIP (**d**) confirms multiple “blooming” hypointense foci related to blood products. Sagittal T1-weighted images before (**e**) and after (**f**) contrast injection show spontaneous hyperintensity and patchy enhancement of the parieto-occipital lesion
- RT-induced parenchymal injury:
  Leukoencephalopathy, also called “diffuse radiation injury”, demonstrates T2-hyperintensity in the periventricular white matter with sparing of the U-fibers, unlike with Progressive multifocal leukoencephalopathy [11, 13]. Metabolic imaging (such as PET or SPECT) and advanced MRI techniques are required to distinguish recurrent tumors from radionecrosis, in that the enhancing foci reveal decreased rCBV on perfusion, elevated lipid/lactate peaks on MR spectroscopy, and resolution of enhancement on follow-up (Fig. 4) [11].

- RT-induced vascular injury:
  Progressive cerebral arteriopathy with vessel narrowing and irregularity can be observed, sometimes associated with moyamoya disease [13]. Further, RT may induce capillary telangiectasias and cavernous malformations demonstrated as hypointensities on T2/T2*-weighted images (Fig. 3), and mineralizing microangiopathy that can be detected on nonenhanced CT as calcifications in the affected region [11, 13].

**Fig. 4:**
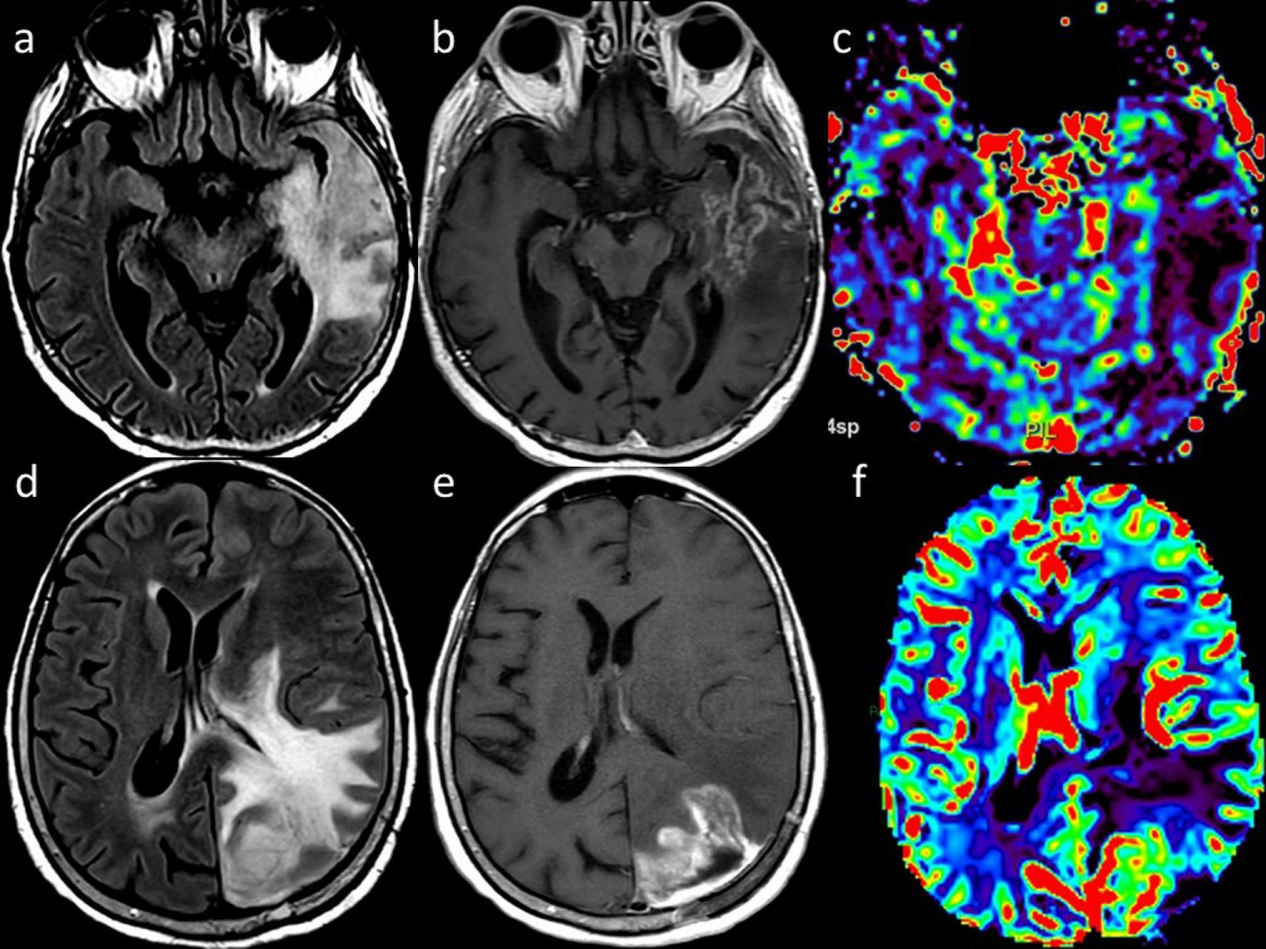
Radionecrosis versus recurrent tumor. Axial FLAIR (**a**), contrast-enhanced T1-weighted (**b**) and DSC rCBV map (**c**) of a 72 year-old man with GBM treated with surgery and ChT/RT show brain swelling, high FLAIR signal, low”soap bubble” enhancement and reduced rCBV in the left temporal lobe, consistent with radionecrosis. Axial FLAIR (**d**), contrast-enhanced T1-weighted (**e**) and DSC rCBV map (**f**) of a 52-year-old woman with glioblastoma treated with surgery and ChT/RT show brain swelling, high FLAIR signal, intense enhancement and increased rCBV, indicative of tumor recurrence
  Transient focal enhancing lesions have been recently described with focal T2-hyperintense lesions and “snowflakes” or curvilinear enhancement in the pons, cerebellum, and posterior cerebral hemispheres [14]. Lastly, it must be pointed out that irradiated patients have sevenfold increased risk of developing secondary brain tumors, specifically meningioma, sarcoma and malignant glioma [11, 14].
- Stroke-like migraine attacks after RT:
  It is an uncommon delayed complication [14]. MRI shows unilateral temporal, parietal, or occipital gyriform T2/FLAIR hyperintensities and enhancement that do not respect vascular territories, while DWI may reveal superimposed infarcts [14].

#### ChT-induced neurotoxicity

**Fig. 5:**
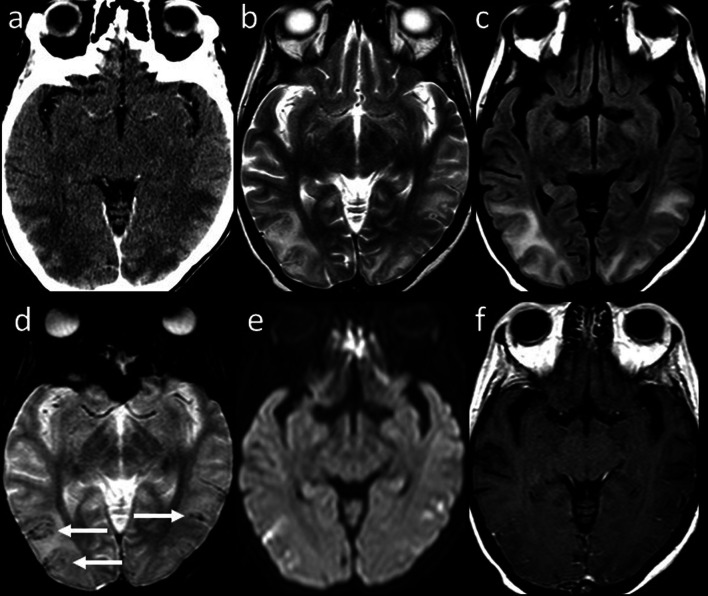
Posterior Reversible Encephalopathy Syndrome. Axial contrast-enhanced CT image (**a**) of a 40-year-old woman with metastatic cervical carcinoma treated with multiple ChT agents including Cisplatin and 5-Fluorouracil shows bilateral parieto-temporo-occipital, subcortical and cortical, hypodense and non-enhancing foci. Axial T2-weighted (**b**) and FLAIR (**c**) images reveal corresponding hyperintense areas. Axial GRE T2*-weighted (**d**) image shows cortical and focal hypointense foci (arrows) related to microhemorrhages with neither diffusion abnormality on DWI (**e**) nor pathologic contrast enhancement on axial post-contrast T1-weighted image (**f**)

Although the ever-growing number of new ChT agents, most drugs tend to produce similar patterns of injury on MRI, or present pathognomonic findings (i.e., ipilimumab-induced hypophysitis). Acute or chronic leukoencephalopathy may occur after administration of methotrexate, 5-fluorouracil and fludarabine, and is demonstrated on MRI as bilateral involvement of subcortical and periventricular white matter with T2/FLAIR hyperintensities showing diffusion restriction but lack of contrast enhancement [12]. Posterior reversible encephalopathy syndrome can be caused by several drugs (e.g., cisplatin, cyclophosphamide, methotrexate, bortezomib, sorafenib, rituximab, bevacizumab, Immune Checkpoint Inhibitors (ICIs), and Chimeric antigen receptor T cell). MRI typically shows symmetric subcortical T2-hyperintensities involving occipital and parietal lobes without diffusion restriction (Fig. 5) [11, 13].

Cerebrovascular disorders may be either ischemic or hemorrhagic (i.e., after antiangiogenic drugs, such as bevacizumab); L-asparaginase may lead to venous sinus thrombosis [12].

Cerebellar syndrome has been reported in patients undergoing cytosine arabinoside or, more rarely, 5-fluorouracil. In these patients, MRI can be initially unremarkable, followed by development of cerebellar atrophy within months after treatment [12]. Myelopathy is a rare complication of intrathecal ChT with methotrexate, cytosine arabinoside, and nelarabine [12].

Beyond classic chemotherapeutic drugs, ICIs, such as ipilimumab, nivolumab, and pembrolizumab, enhance immune antitumoral activity and may lead to complications including autoimmune hypophysitis, limbic encephalitis, meningoencephalitis, and cerebellitis, while rare manifestations include posterior reversible encephalopathy syndrome and transverse myelitis [12]. On MRI, classic ipilimumab-induced hypophysitis appears as diffuse enlargement of the pituitary gland, with variable thickening of the infundibulum [15].

### Head and neck

Several treatment regimens for head and neck (HN) cancers involve RT alone or in combination with ChT as either a radical or adjuvant postsurgical treatment [16, 17]. Concurrently with the expected curative results on the tumor, these treatments induce a variety of side effects on normal tissues neighboring the neoplasm, presenting as inflammatory/reactive changes or clinically relevant complications.

The tissue modification process generally starts alongside the radiation exposure; notwithstanding, the irradiated tissue may show clinically and radiologically evident abnormalities even months or years later [18]. Actively proliferating tissues show early modifications more often than tissues with a slow replication, which will be typically interested by delayed changes [19].

**Fig. 6:**
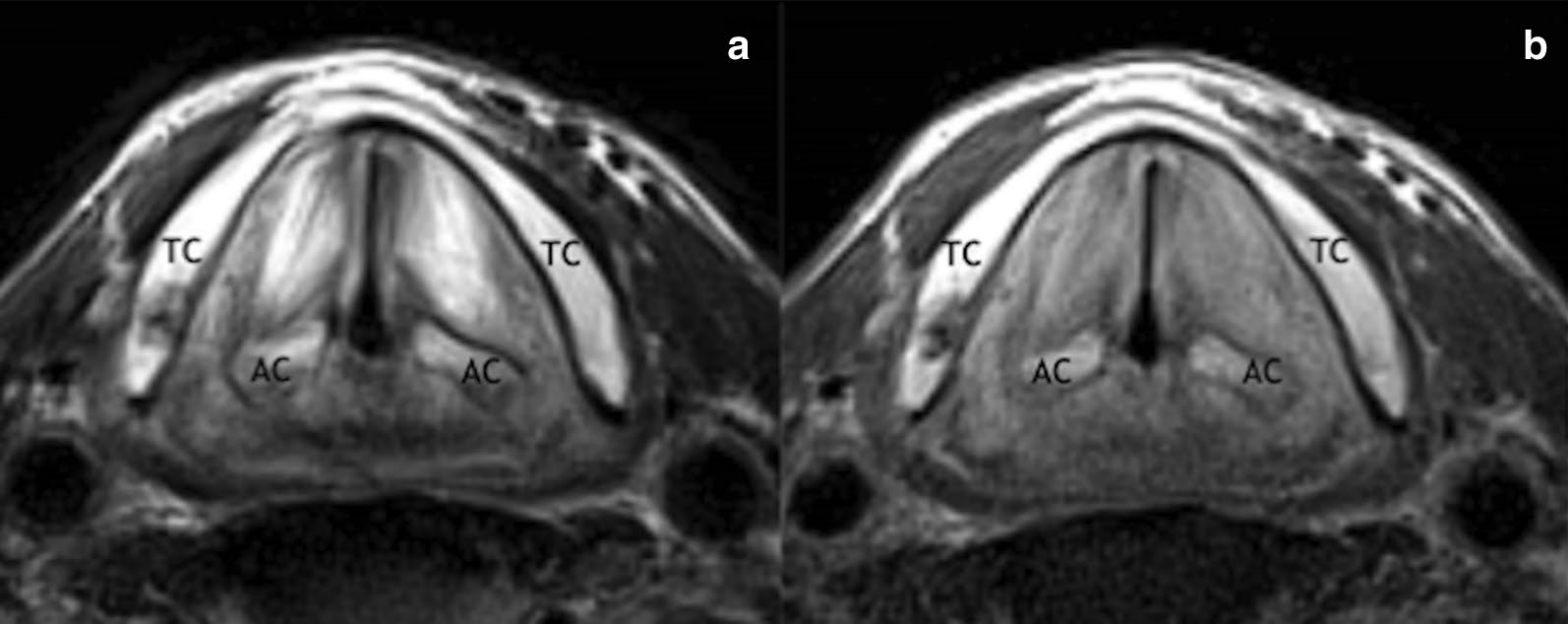
Chronic edema of the true vocal folds 14 years after ChT/RT in a 72-year-old man with lymph node metastasis from unknown primary. Axial T2-weighted (**a**) and T1-weighted (**b**) images acquired with surface-coil MRI of the larynx show diffuse T2-hyperintensity and slightly T1-hyperintensity of the true vocal folds. TC, thyroid cartilage; AC, arytenoid cartilage

**Fig. 7:**
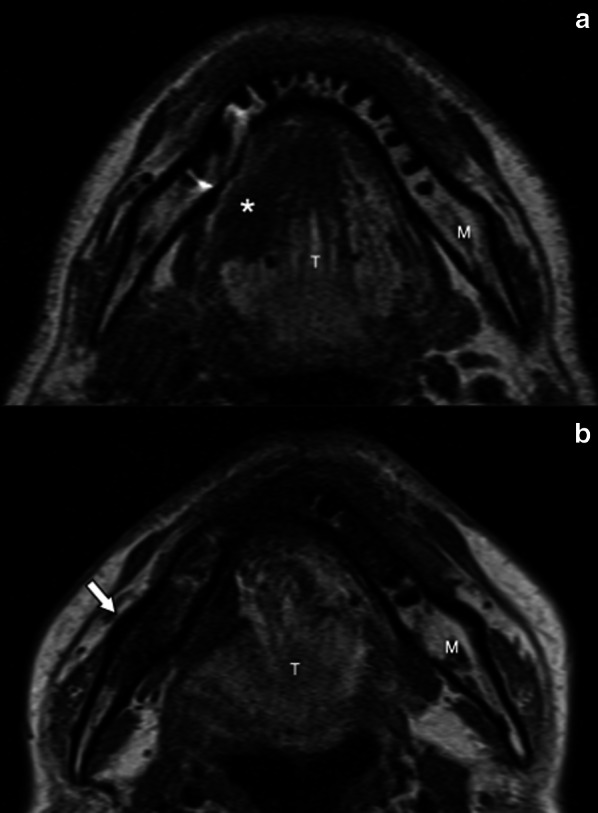
Osseous sclerosis of the right ramus of the mandible (arrow) in a 44-year-old man 5 years after right hemiglossopelvectomy followed by adjuvant ChT/RT for a carcinoma of the right border of the tongue. Pretreatment axial T1-weighted (**a**) MR image shows a lesion of the right border of the tongue (asterisk) and normal signal intensity of the right ramus of the mandible. Post-treatment axial T1-weighted (**b**) MR image shows the surgery outcome and diffuse hypointense signal of the spongiosa of the right ramus of the mandible (arrow). M, mandible; T, tongue

**Fig. 8:**
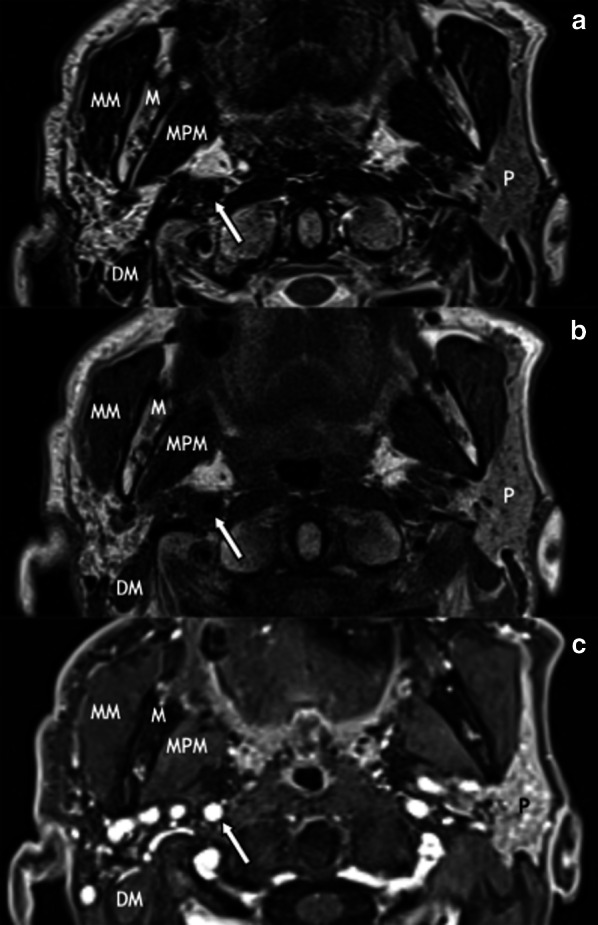
Atrophy of the right parotid gland in a 73-year-old man 3 years after ChT/RT for a carcinoma of the right inferior alveolar crest. MR images show a right parotid gland smaller than the contralateral, with high signal on axial T2-weighted (**a**), high signal on T1-weighted (**b**), and low signal on post-contrast 3D GRE fat-sat T1-weighted (**c**) images, due to extensive fatty infiltration of the parenchyma. P, parotid gland; M, mandible; MM, masseter muscle; MPM, medial pterygoid muscle; DM, digastric muscle; internal carotid artery (arrow)

There are some expected RT-related changes that are related to a persistent inflammatory process:

- thickening of the superficial soft tissues (skin and platysma muscle);
- subcutaneous fat reticulation;
- deep fat tissue reticulation and edema of the deep anatomical spaces;
- swelling, edema, and enhancement of mucosa and muscles (especially laryngeal, pharyngeal and masticator muscles) (Fig. 6);
- osseous sclerosis (Fig. 7);
- increased enhancement of the major salivary glands and thyroid, followed by size reduction and alteration of their structure (Fig. 8).

**Fig. 9:**
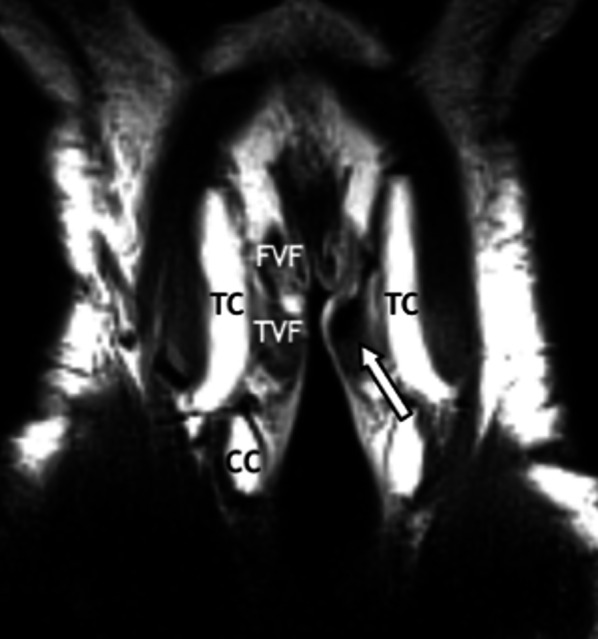
Fibrosis of the left true vocal fold (arrow) in a 60-year-old man 3 years after ChT/RT for laryngeal cancer. Coronal T2-weighted MR image acquired with surface-coil MRI of the larynx shows hypointense signal of the left true vocal fold. TC, thyroid cartilage; CC, cricoid cartilage; FVF, false vocal fold; TVF, true vocal fold

The swelling of the superficial soft tissues and muscles due to the acute inflammatory process may evolve in the long term to fibrosis with subsequent induration and functional impairment (Fig. 9) [20, 21].

High radiation doses and large radiation fields, as well as persistent smoking habits and alcohol abuse, may facilitate the onset of uncommon post-RT complications [21].

**Fig. 10:**
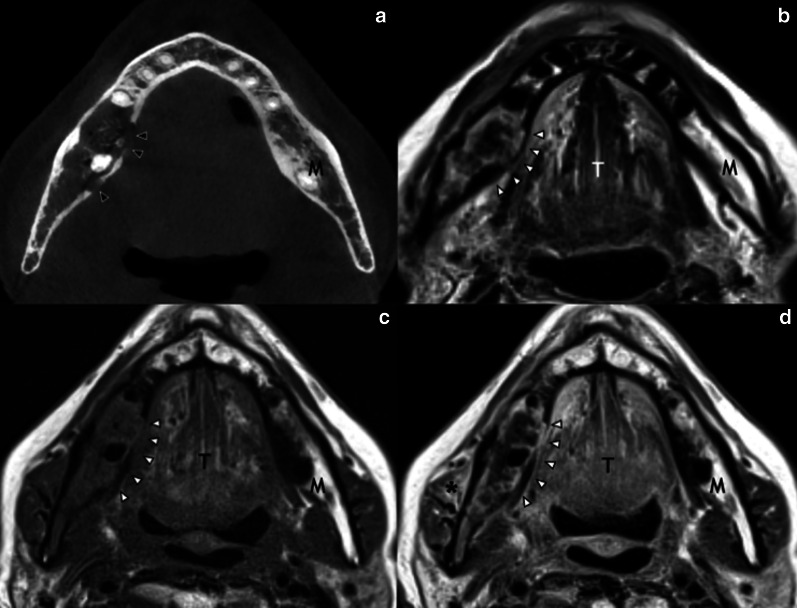
Osteoradionecrosis of the right ramus of the mandible in a 70-year-old woman 4 years after ChT/RT for a right palatine tonsil cancer. Axial CT image (**a**) shows the loss of spongiosa trabeculation and several cortical breakings (black arrowheads) of the right ramus of the mandible. The corresponding MR slice of the same area (white arrowheads) shows heterogeneous and slightly hyperintense signal on axial T2-weighted (**b**), with low signal on axial T1-weighted (**c**), and subtle but diffuse enhancement in the bone and adjacent soft tissues, like the right masseter muscle (asterisk), in post-contrast axial T1-weighted image (**d**). M, mandible; T, tongue

**Fig. 11:**
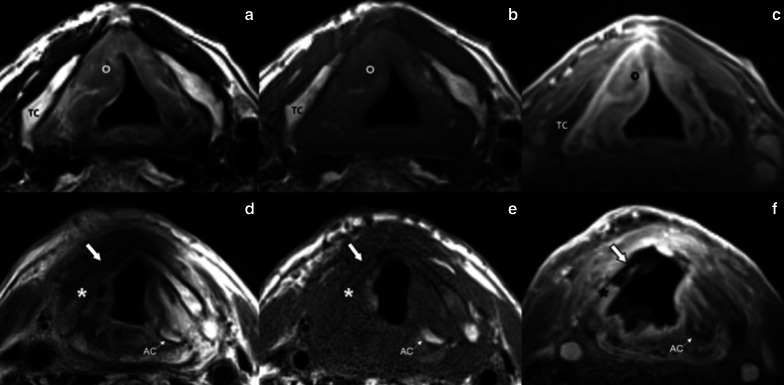
Laryngeal necrosis in an 88-year-old man five months after RT for a right glottic-supraglottic cancer. Surface-coil MRI of the larynx: pre-treatment axial T2-weighted (**a**), T1-weighted (**b**) and post-contrast 3D GRE fat-sat T1-weighted (**c**) MR images show a lesion of the right vocal fold (circle). Post-treatment axial T2-weighted (**d**), T1-weighted (**e**) and post-contrast 3D GRE fat-sat T1-weighted (**f**) show extensive necrosis of the right true vocal fold, anterior commissure and right lamina of the thyroid cartilage (asterisk); the right arytenoid cartilage is not recognizable. Cartilage fragmentation is pointed by the bold white arrow. TC, thyroid cartilage, AC, arytenoid cartilage

- Mucosal necrosis: most necroses occur within the first 2 years post-RT, with a peak between 6 and 12 months [21]. On cross-sectional imaging, mucosal necrosis should be suspected when mucosal enhancement is absent; superficial ulceration can also be associated. Gas pockets are frequently seen around the necrotic area [21, 22].
- Osteoradionecrosis: it usually occurs 1–3 years post-RT, with a reported incidence between 0% and 37.5% [21]. The most affected site is the mandible, probably due to a relatively limited vascularization and superficial location. The most frequent bone abnormalities revealed by CT are interruptions of the cortical margins and loss of trabeculation of the spongiosa, commonly associated with pathological fractures. Soft tissue swelling and enhancement (sometimes with gas bubbles) can be seen both on CT and MRI surrounding the site of osteonecrosis. MRI shows also bone marrow signal changes with T1-hypointense, heterogeneous slightly T2-hyperintense areas, and strong and inhomogeneous contrast enhancement (Fig. 10) [20, 21, 23].
- Chondronecrosis: it is usually consequent to post-treatment infectious perichondritis and may lead to cartilage fragmentation and intraluminal collapse. In laryngeal necrosis, cartilaginous abnormalities are visible on CT often with laryngeal soft tissues swelling and possible fluid and gas bubbles surrounding the cartilage itself. On MRI, it is revealed by T1 hypointensity and inhomogeneous gadolinium enhancement of the medullary space of ossified cartilage; the cortical rim shows irregular borders and focal fractures. Inflammatory changes of the surrounding soft tissues are common (Fig. 11) [20].
- Vascular complications: accelerated atherosclerosis of the carotid arteries is a well-known effect of radiation which often complicates preexisting vascular disease. Pseudoaneurysm formation of the internal carotid artery is a life-threatening but rare complication. Thrombosis of the internal jugular vein may also occur [21].
- Trismus: it is most likely related to pterygoid muscle and temporomandibular joint fibrosis, with a peak at 12–18 months post-RT. MRI shows T2 hyperintense downsized muscles with enhancement characterized by a linear edge corresponding to the radiation field [20].
- Radiation-induced neoplasms: they arise within the radiation field at least 3 years after RT, with a different histology compared to the primary tumor [24]. Most frequent malignancies are squamous cell carcinomas, thyroid tumors, sarcomas, and lymphomas.

### Lung

Radiation-induced lung injury (RILI) is a well-known event after RT for lung and breast cancer. In this setting, hypofractioned RT has shown to be effective and better tolerated treatment approach in lung cancer patients not suitable of concurrent RT/ChT [25, 26]. Several cellular and molecular changes occur in lung tissue after RT, with inflammatory cells infiltration and production of reactive nitrogen and oxygen species that determine oxidative damage of DNA and epithelial cell death leading to RILI [27, 28]. RILI is a significant cause of reduced quality of life in cancer survivors and presents with two distinct clinical phases: early (< 6 months) reversible adverse effects in the form of pneumonitis and late adverse fibrotic effects [29, 30]. Early CT changes include diffuse consolidation, patchy consolidation, diffuse ground-glass opacities (GGOs), patchy GGO, or no evidence of increasing density. Concerning late fibrotic changes, four categories of CT features may be observed: modified conventional pattern of fibrosis (consolidation, volume loss, bronchiectasis ± GGO), mass-like fibrosis (well-circumscribed focal consolidation limited to the tumor region but which is larger than the original tumor), scar-like fibrosis (a linear opacity in the tumor region with associated volume loss), or no evidence of increasing density [27]. A particular form of RILI is radiation-induced bronchiolitis obliterans organizing pneumonia, a clinico-pathological entity in the wide family of pulmonary fibrosis characterized by patchy and often migratory peripheral air space infiltrates of the lungs outside of the radiation field, unlike radiation pneumonitis [31].

ChT agents may induce lung injury by various mechanisms including direct toxic effect on the lung, immunologic response, and increase in capillary permeability. Timing of clinical manifestations is variable ranging from early onset to years after the beginning of ChT [32]. Further, there is no consensus on the duration of therapy-induced changes, which is extremely variable. Remission can be reached with dose reduction, drug withdrawal and associated administration of steroids, with evidence suggesting relapse if steroids are stopped early (within 3 months of onset) [32]. Notably, ChT-induced effects are not related with the duration of treatment, but these are frequently dose related. Patients with impaired renal function may experience increased drug accumulation and are at risk of increased lung toxicity. Conversely, hypersensitivity-type reactions are frequently dose independent. ChT-induced lung disease can present with interstitial pneumonitis/fibrosis and cryptogenic organizing pneumonia, hypersensitivity syndrome, hemorrhage, and capillary leak syndrome [33]; less common findings are pleural effusions, bronchospasm, hilar adenopathy, and veno-occlusive disease [34]. Capillary leak syndrome is characterized by an increase in vascular permeability, causing extravasation of fluids and proteins from capillary vessels into the soft tissues and resulting in interstitial edema. Capillary leak syndrome, and in general non-cardiogenic pulmonary edema due to lung toxiticy, is characterized by the presence of severe hypoxemia, bilateral alveolar infiltrates, GGOs, thickened septal lines, often with no pleural effusion and no evidence of left atrial hypertension/congestive heart failure [35].

**Fig. 12:**
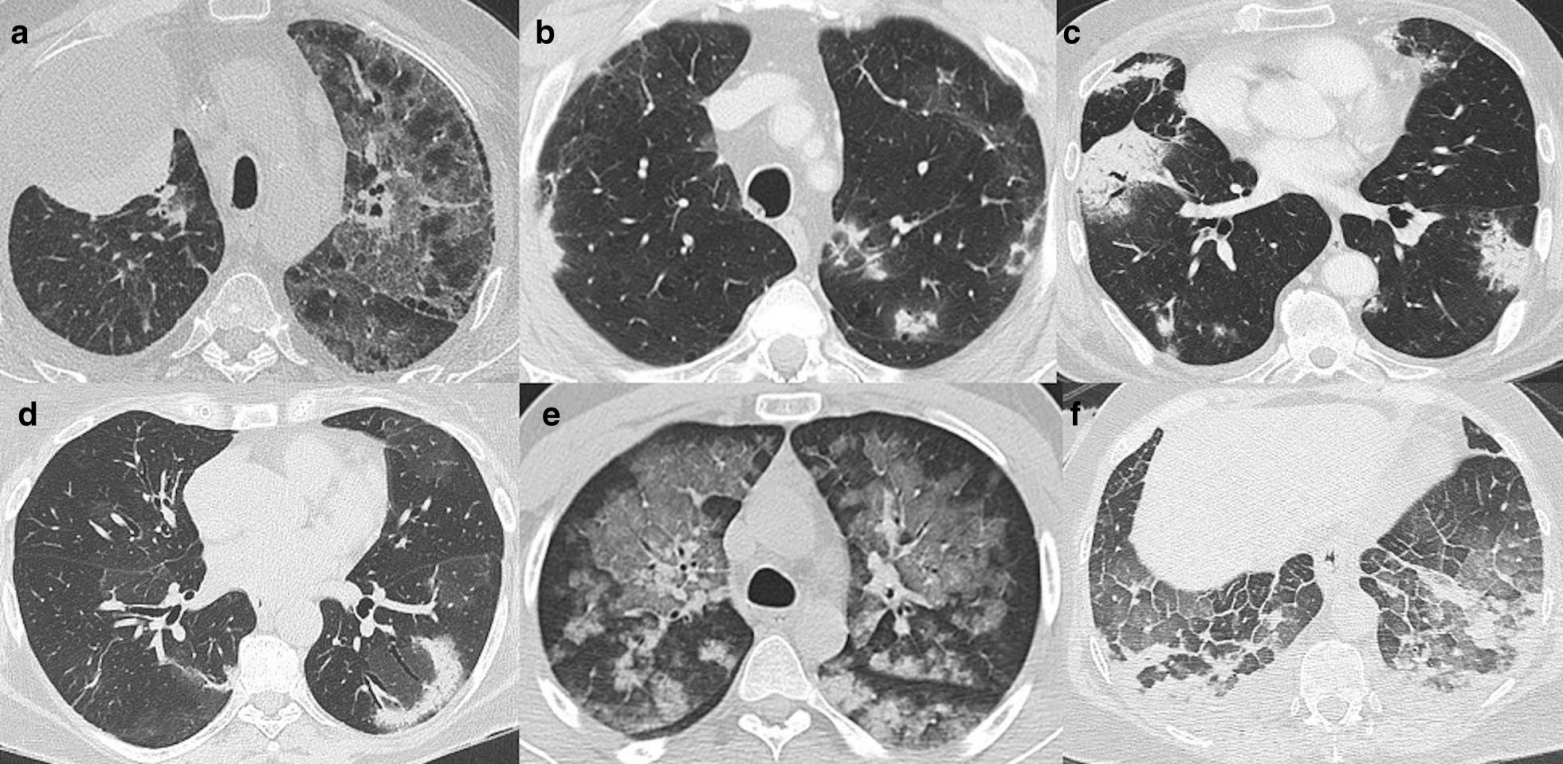
Axial CT images of different cases of ChT/RT-related lung toxicity. **a** shows acute pneumonitis after the first administration of an anti-PD-1 target immunotherapic agent (Pembrolizumab) in a patient with right lung cancer. Also Nivolumab (another anti-PD-1 agent) can cause lung adverse disease with interstitial pneumonitis presenting with organizing pneumonia pattern, as shown in (**b**, **c**). An example of radiation-primed cryptogenic organizing pneumonia is reported in (**d)**, which shows the so-called “reverse halo sign” or “atoll sign” in the left lower lobe after RT for breast cancer. **e** Shows acute and diffuse alveolar hemorrhage in a patient with NSCLC treated with Bevacizumab, with bilateral GGOs caused by blood filling alveolar spaces. Acute lung edema with bilateral smooth lobular septal thickening and GGOs in a patient treated with IL-2 is shown in (**f**)

Bevacizumab is among drugs that can induce lung hemorrhage, while gemcitabine and immune-mediated therapies such as interleukin 2 and interferon may lead to ARDS or noncardiogenic pulmonary edema [33]. Nevertheless, bleomycin must be mentioned among the most important ChT agents that induce lung disease; several radiologic patterns have been associated with this drug including cryptogenic organizing pneumonia, eosinophilic hypersensitivity, and interstitial pneumonitis [33]. Radiologists need to know imaging features of RILI and ChT-induced lung disease given that early detection of these conditions may be crucial to reduce the extent of lung damage. Figure 12 shows some examples of ChT/RT-related lung toxicity.

### Cardiac

Cardiotoxicity is one of the greatest challenges in the management of oncology patients, as it can compromise the success of treatment. Current RT techniques allow for focused radiation beams, sparing organs at risk thereby minimizing direct cardiac dose, with subsequent reduction of RT-related cardiac complications [36, 37]. Unfortunately, adverse events associated to ChT have been recorded not only with older ChT drugs (e.g., anthracyclines), but also with recently introduced immunotherapies and targeted therapies. In Table 2 the most frequent imaging scenarios of myocardial injury related to RT and ChT toxicity are summarized.

**Table 2:** Most common RT and ChT-induced myocardial damage

Treatment	Pathophysiology	Myocardial disease	Coronary CT	Cardiac MRI
RT	Endothelial injury and inflammation leading to endothelial dysfunction and acceleration of the atherosclerotic process in the coronary vessels and chronic collagen deposition lead to fibrosis of soft tissues	CAD	CAD	Perfusion defect in case of obstructive coronary artery disease
		Valvular disease	Diffuse thickening of leaflets with calcifications in late stages	Fibrotic thickening and enhancement of leaflets with dysfunction (stenosis or regurgitation) in late stages
		Constrictive pericarditis	Increased pericardial thickness Pericardial calcifications	Increased pericardial thickness with delayed enhancement Pericardial calcifications Septal flattening or bounce Inspiratory septal shift
		Cardiomyopathy	Non-obstructive CAD	Increased ECV for fibrosis 10% decline of EF or EF < 50%
ChT	Mitochondrial dysfunction, inflammation, oxidative stress, with cardiomyocyte apoptosis and necrosis, and replacement fibrosis (with reported dose-dependent mechanism)	Myocarditis (early toxicity)	Non-obstructive CAD	Slightly altered T2 mapping and ECV with or without preserved EF
		Cardiomyopathy	Non-obstructive CAD	Increased ECV for diffuse fibrosis 10% decline of EF or EF < 50% in late and irreversible stage
ICI	The pathological mechanism is not fully understood, it seems to be related to hyperactivation of the immune system Histological evidences of upregulation of inflammatory cytokines and of massive inflammatory cell infiltration	Pericarditis	Pericardial thickening and enhancement Pericardial effusion	Pericardial thickening, edema and enhancement Pericardial effusion
		Myocarditis	Non-obstructive CAD	Diffuse myocardial edema, increased native T1, T2 and ECV LGE with non-ischemic pattern
		Other myocardial diseases (rare)	Non-obstructive CAD	Stress cardiomyopathy (rapid EF recovery) and Takotsubo-like cardiomyopathy Ischemic LGE for myocardial infarction (due to vasospasm)

RT, radiation therapy; ChT, chemotherapy; ICI, immune checkpoint inhibitors; CAD, coronary artery disease; EF, ejection fraction; T1, native T1 mapping; ECV, extracellular volume fraction; LGE, late gadolinium enhancement

RT was found to induce endothelial dysfunction causing microvascular disease. Indeed, preclinical studies showed that RT-induced endothelial dysfunction determined microvascular damage with reduced capillary density and ischemia, which led to subsequent inflammation, cardiomyocyte loss, fibrosis, and ventricular dysfunction [36]. RT affects also the biological pathway of age-related atherosclerosis with an acceleration of atherosclerotic process. As a proof, Darby et al. [38] reported a close association between the mean heart dose and cardiac events with a significant increase of 7.4% in the rate of acute coronary events per 1 Gy. Further, older age at first RT resulted associated to higher risk of cardiac events and earlier onset [36].

Coronary-CT is the imaging of choice for coronary artery disease characterization and prognostication. In agreement with preclinical studies reporting an acceleration of atherosclerosis, the history of cancer and treatments has been markedly associated with increased coronary artery calcium scores compared to the general population in a 10-year follow-up study [39]. In line with this finding, elevated coronary artery calcium scores have been found after RT in Hodgkin’s lymphoma survivors, making coronary-CT and coronary artery calcium scores potential imaging tools to evaluate RT-related coronary artery disease [40]. As a matter of fact, mediastinal RT has shown to increase 6.8 times the risk of coronary artery abnormalities in Hodgkin’s lymphoma patients if compared to those not subjected to RT [41]. Moreover, high-dose chest RT was found to determine valve stenosis and constrictive pericarditis, which are late sequalae that become clinically evident 10–20 years after RT. Indeed, valve stenosis has been reported in 5–32% patients treated for Hodgkin lymphoma and can be noninvasively assessed also through imaging [42].

**Fig. 13:**
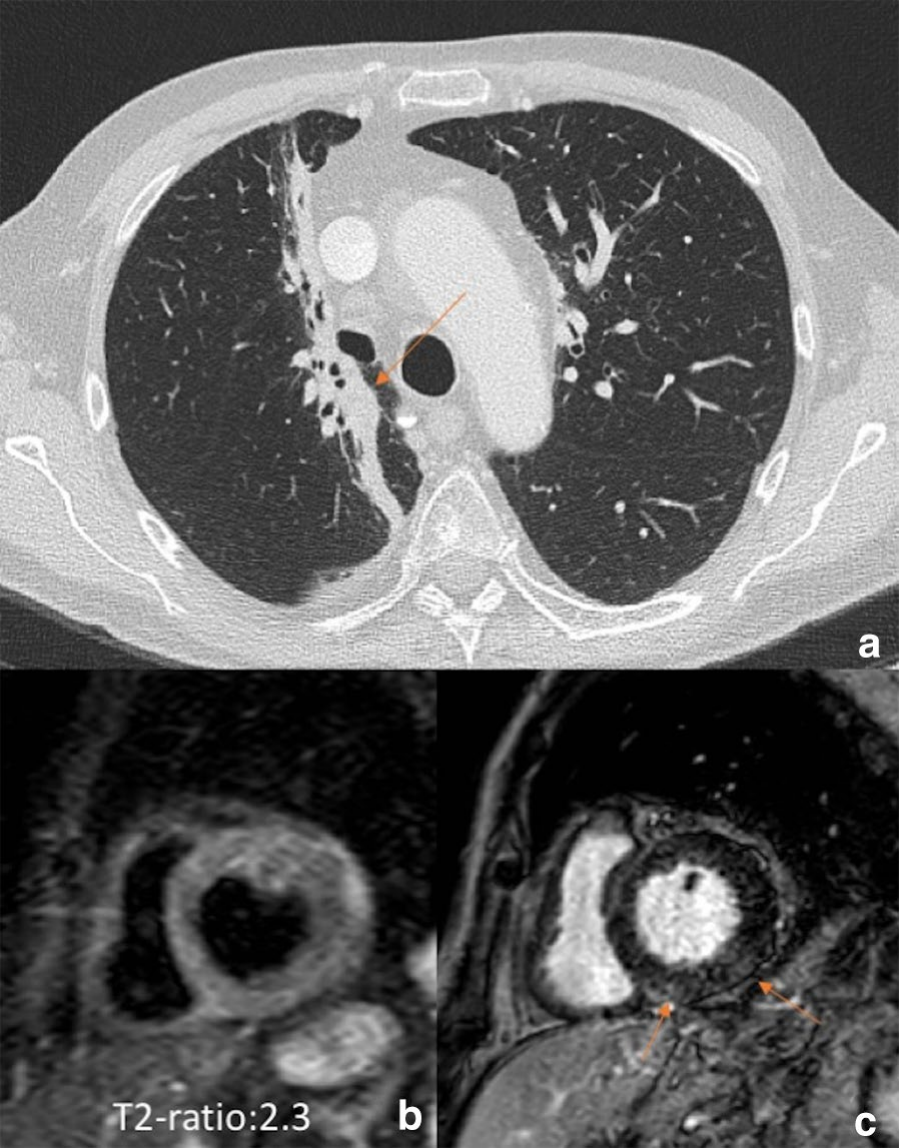
Myocarditis associated to immune checkpoint inhibitor in a patient with lung cancer. A 67-year-old man with advanced lung adenocarcinoma (stage IIIB) was initially treated with ChT/RT. Axial CT image (**a**) showing right lung RT-related changes around the primitive cancer (arrow). Due to an incomplete response, treatment with immune checkpoint inhibitor was administered. The patient attended was admitted four months later at the emergency department for acute dyspnea associated to increased high sensitive troponin T (113 ng/L; normal value < 14 ng/L). Cardiac MR showed a left ventricle with normal volume (83 mL/m^2^) but with a slight depression of the ejection fraction (47%) associated to a diffuse hyperintensity on STIR image and increased T2-ratio (**b**) suggestive for diffuse edema and a shadowed late gadolinium enhancement in the inferior wall with non-ischemic pattern. MR findings indicated active myocarditis which was confirmed at endomyocardial biopsy

The spectrum of ChT-related cardiac complications is wide and differ depending on the drug, all of them leading to ventricular dysfunction. Pathological cardiac abnormalities due to cancer treatments include: (i) accelerated atherosclerosis related to hormone therapy; (ii) mitochondrial dysfunction with cell death and fibrosis due to anthracycline; (iii) coronary artery disease, ischemia, thrombosis and hypertension due to vascular endothelial growth factor inhibitor, tyrosine kinase inhibitors and antimetabolites; and (iv) myocarditis with cardiogenic shock and life-threatening ventricular arrhythmias due to ICIs [43]. Given that the effect of ChT-induced myocardial injury mostly converge in ventricular dysfunction and heart failure, current guidelines for surveillance and detection of cardiotoxicity recommended monitoring cardiac function before, during and after treatment with the estimation of three-dimensional ejection fraction and global longitudinal strain [43]. Three-dimensional echocardiography is the first line imaging choice to assess ejection fraction and volumes with good accuracy and reliability, wide availability, cost-effectiveness and lack of radiation exposure. However, preserved ejection fraction does not exclude significant myocardial injury. For this reason, noninvasive imaging biomarkers to improve early detection of cardiotoxicity have been recently investigated [43–45]. Cardiac MRI is the non-invasive gold standard for morpho-functional myocardial characterization, being able to provide accurate volume and function estimation and information about tissue inflammation/edema and fibrosis, therefore improving the detection of cardiotoxicity over conventional functional assessment. Cardiac MRI can identify ChT-related microstructural changes including myocyte loss through the estimation of the intracellular and extracellular volume fraction. In particular, extracellular volume fraction resulted able to depict drug-induced extracellular matrix remodeling and edema. Indeed, extracellular volume fraction is sensitive to extracellular volume expansions related to edema in acute stages or diffuse fibrosis in later stages. These findings have the potential to improve clinical management of oncology patients, helping to detect cardiac damage in reversible stage. Nevertheless, the routine use of cardiac MRI is not currently recommended due to limited availability and high cost. The only exception is for patients receiving ICIs therapy in order to exclude treatment-related myocarditis (Fig. 13) [43], which is relatively uncommon (< 1%), but potentially life threatening (approximately 40% of death rate)[45].

### Liver

ChT, RT, or their combination may cause liver injury related to both a direct hepatic toxicity and a non-hepatic toxicity due to an altered hepatic clearance.

**Fig. 14:**
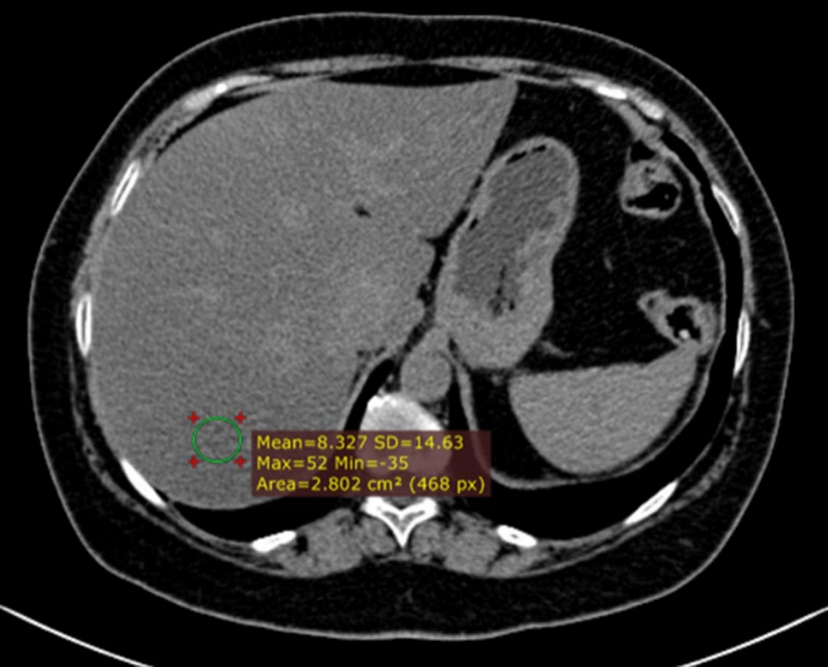
59-year-old woman with history of hysterectomy, bilateral salpingo-oophorectomy and segmental resection of distal left ureter for uterine leiomyosarcoma, and tumor relapse at first CT follow-up after 3 months from surgery and then treated with chemotherapy (doxurubicine-dacarbazine). Precontrast CT at 3 months after chemotherapy shows low attenuation of the liver parenchyma with an absolute value of 8 HU, consistent with post-ChT steatosis

**Fig. 15:**
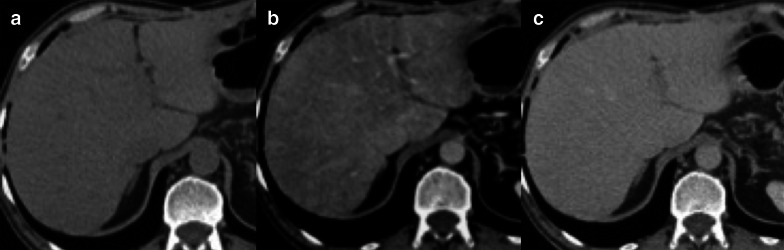
64-year-old man with history of left hemicolectomy for colon carcinoma and liver metastases treated with oxaliplatin. Contrast enhanced CT on precontrast (**a**), portal venous (**b**) and delayed (**c**) phases demonstrates a patchy liver enhancement with a mosaic appearance on portal venous phase, mainly in the periphery of the right lobe, with normal parenchymal enhancement on subsequent delayed phase. These findings are consistent with oxaliplatin-induced sinusoidal obstruction syndrome in its diffuse form

**Fig. 16:**
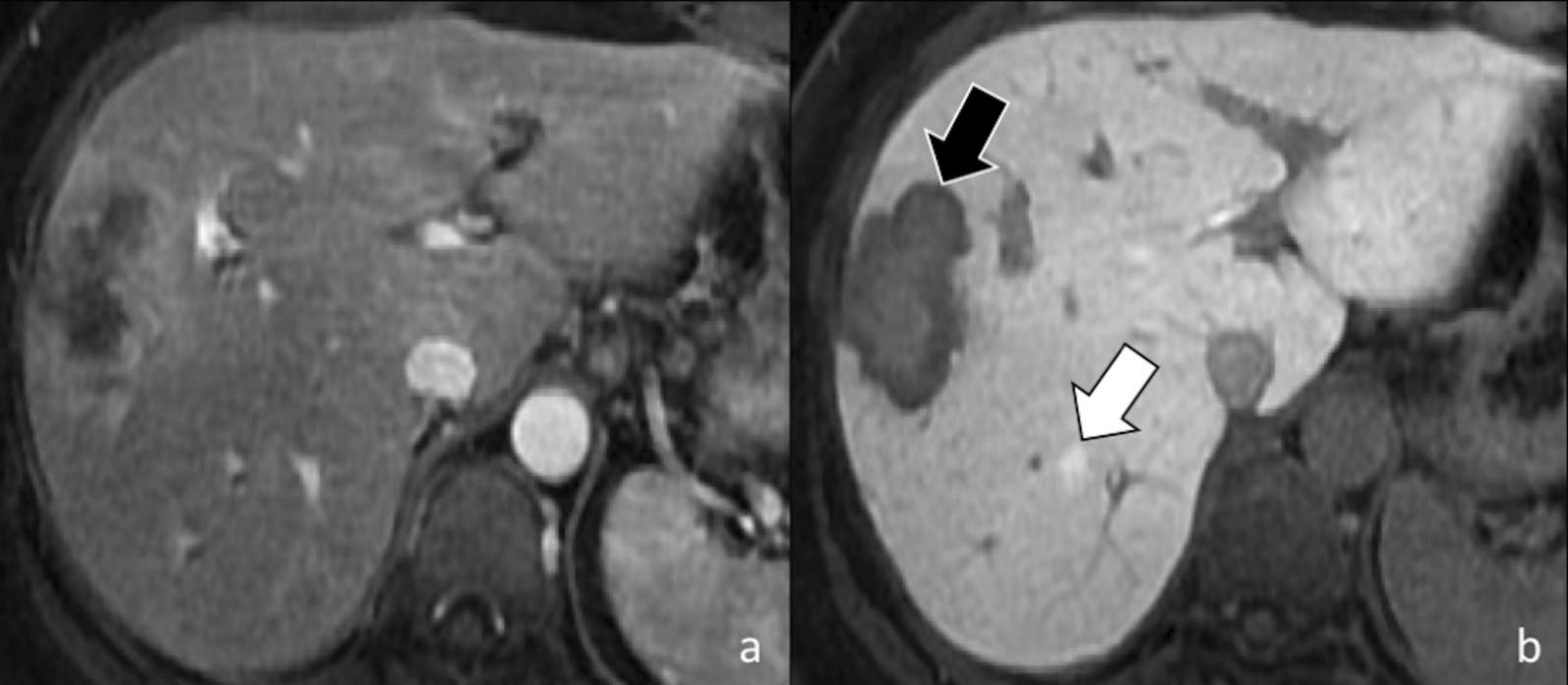
44-year-old woman with breast cancer and liver metastasis treated with chemotherapy. Gadoxetate disodium-enhanced MRI shows a 7 mm regenerative nodule (white arrow) that is not visible in the arterial phase (**a**), and shows contrast retention in the hepatobiliary phase (**b**). In the same patient a liver metastasis (black arrow) shows peripheral rim enhancement in the hepatic arterial phase (**a**) and a target appearance in the hepatobiliary phase (**b**) with central hyperintensity due to contrast retention and a peripheral hypointense rim

ChT has been widely used for years in the management of primary or secondary liver tumors. The different mechanisms of action of ChT may result in a broad spectrum of clinical, pathological and radiological hepatic injuries, including diffuse and/or focal hepatopathy, such as acute or chronic hepatitis, steatosis, fibrosis, pseudocirrhosis, sinusoidal changes (i.e., sinusoidal obstruction syndrome [SOS], centrilobular sinusoidal dilatation or peliosis) and nodular hyperplasia (Fig. 14) (i.e., nodular regenerative hyperplasia or FNH-like lesions). The main ChT-induced hepatopathy with corresponding CT and MRI findings are presented in Table 3 [46]. Acute hepatocellular injury does not have specific imaging findings, and it is hypothesized that it may be detected during the hepatobiliary phase as impaired hepatocellular uptake of gadoxetate disodium. Chronic hepatocellular injury may lead to focal or diffuse morphological changes, such as confluent fibrosis and pseudocirrhosis, respectively. Imaging detection of pseudocirrhosis following ChT with severe portal hypertension may lead to interruption or change of the ChT drug to avoid major complications. ChT-induced steatosis and SOS (Fig. 15) are amongst the most common occurring in about 47% and 77.4% of patients, respectively [47, 48]. Steatosis may be easily depicted on cross-sectional imaging as low attenuation of liver parenchyma on CT (Fig. 16) (i.e. liver attenuation lower than 40 HU or liver attenuation lower than 10 HU as compared to the spleen) or signal loss in the opposed phase as compared to the in-phase images on MRI. The diagnosis of SOS includes indirect signs related to reduced liver outflow and portal hypertension (e.g. ascites, gallbladder wall thickening, splenomegaly) as well as a direct demonstration of patchy liver enhancement with a mosaic appearance mainly at the periphery of the right lobe on contrast-enhanced CT and MRI and reticular hypointensity on hepatobiliary phase at gadoxetate disodium-enhanced MRI [46]. Less frequently, SOS may be focal and thus its hypointensity on hepatobiliary phase may mimic a metastasis; however, focal SOS lacks diffusion restriction on diffusion-weighted imaging (DWI), which is helpful for the differential diagnosis with metastases [46]. Post-ChT occurrence of FNH-like lesions deserves also attention because the strong arterial phase hyperenhancement of these lesions and their appearance on CT/MRI performed during follow-up in oncology patients may induce to a false diagnosis of metastasis. However, the typical iso- to hyperintensity or doughnut-like appearance of FNH-like lesions in the MRI hepatobiliary phase allows a confident diagnosis [49]; more recently, MR elastography has proved to be even more accurate than DWI for differentiating benign and malignant focal liver lesions [50], and, therefore it may be considered to improve a non-invasive differential diagnosis. Finally, it is also known that ChT-induced liver steatosis may prevent the detection of liver metastases on CT [46].

**Table 3:** Most common ChT-induced parenchymal and vascular hepatic changes

Type of liver injury	Main CT/MRI findings
Acute hepatocellular injury	Hepatosplenomegaly, gallbladder wall thickening, reduced and heterogeneous liver enhancement, abdominal ascites, reduced portal flow, periportal edema
Chronic hepatocellular injury	Capsular retraction, confluent fibrosis, pseudocirrhosis (i.e., liver surface nodularity, multifocal capsular retraction, decreased liver size, enlargement of the caudate lobe, signs of portal hypertension)
Nonalcoholic fatty liver disease	Hepatic steatosis (lower attenuation of the hepatic parenchyma on CT, signal drop on opposed phase compared to the in-phase images on MRI)
Sinusoidal obstruction syndrome	Diffuse form: patchy liver enhancement with a mosaic appearance, mainly in the periphery of the right lobe; focal form: lack of rim-enhancement in the arterial and portal venous phases, lack of diffusion restriction on DWI, intermingled hypointensity and ill-defined margins in the hepatobiliary phase
FNH-like lesions	Usually multiple; homogenous arterial contrast enhancement and isoattenuation to the surrounding liver parenchyma in the portal venous and delayed phase; on hepatobiliary phase MRI iso or hyperintense or with a ring or doughnut-like appearance (i.e., hyperintense periphery and central hypointensity)

**Fig. 17:**
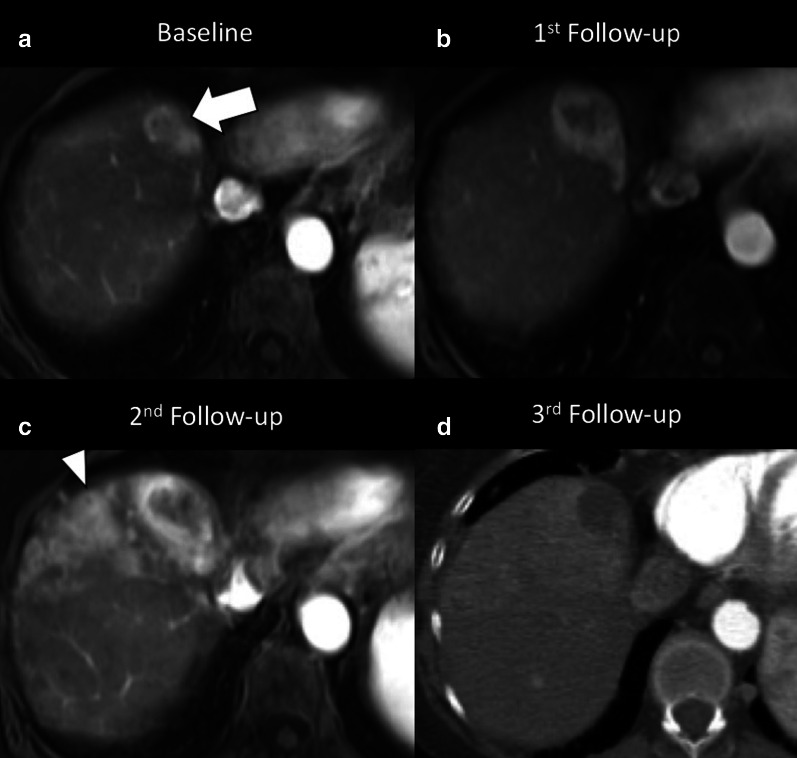
65-year-old woman with viral cirrhosis and hepatocarcinoma previously treated with transarterial embolization with post-treatment persistence of viable tumor (LR-TR viable), then submitted to stereotactic body RT with radiation dose of 40 Gy in 5 fractions. **a** Contrast-enhanced MRI performed before stereotactic body RT shows the hypervascular HCC (arrow) on arterial phase. **b** Contrast-enhanced MRI performed two months after treatment showed a size increase of the lesion with reduced enhancement in the central area. **c** Contrast-enhanced MRI performed four months after stereotactic body RT shows an ill-defined peritumoral enhancement consistent with radiation induced liver disease, that prevents from adequate assessment of tumor margins; the lesion itself shows greater central hypointense area with peripheral enhancement. **d** Contrast-enhanced CT performed 6 months after stereotactic body RT shows a markedly reduced enhancement of the tumor, as well as reduction of peritumoral enhancement. Therefore, the tumor increase in size in the first follow-up at 2 months was not a true progressive disease and was likely related to RT-related changes. *Case courtesy of Dr. Daniele Marin, Duke University Medical Center, Durham (NC), USA*

The adoption of RT for liver lesions has been limited for years as a result of normal tissue tolerance concerns. However, in the last decade, modern RT regimens, including stereotactic body RT and yttrium 90 transarterial radioembolization, have attracted increasing attention as therapeutic option for various liver malignancies [51–53]. However, in addition to the desired effect on tumors, RT exposes to a variable radiation dose and may lead to focal or diffuse changes on healthy liver parenchyma known as radiation-induced liver disease in case of external radiation or radioembolization-induced liver disease in patients treated with radioembolization [51, 52, 54]. Radiation-induced liver disease from external RT is characterized by symptomatic ascites and elevated liver enzymes but not bilirubin, while radioembolization-induced liver disease presents with ascites, non-elevated transaminases (except for ALP and GGPT), and significant bilirubin increase [55]. Liver changes due to RT-related injury may occur during, within a few weeks or after months or years from RT; it is mainly caused by a veno-occlusive damage due to obliteration of the central vein lumina by erythrocytes with subsequent vascular congestion. In addition, hepatic stellate cells are radiosensitive and their activation after RT may contribute to subsequent development of hepatic fibrosis. As such, common post-treatment imaging findings after RT are peritumoral edema, inflammation, ring enhancement, hepatic fibrosis, and capsular retraction [56]. Peritumoral edema and inflammation are usually the earliest encountered changes after RT and may lead to an apparent increase in tumor lesion size, which could be misinterpreted as progression. As such, in case of transarterial radioembolization, it is recommended to perform tumor response evaluation at least after 3 months from treatment [56]. In regard of the ring enhancement, it is described as a thin rim of enhancement surrounding the treated lesion and it may occur after either stereotactic body RT or yttrium 90 transarterial radioembolization; it is important to know its occurrence as a potential post-treatment change because it may occasionally simulate tumor progression (Fig. 17) [51, 55, 56]. RT may also lead to complications in the nearby organs and structures (e.e. cholecystitis, radiation pneumonitis, RT-induced enteritis) that can be also detected at cross-sectional imaging.

Knowledge of the typical imaging appearance of imaging features of ChT/RT associated liver damage is therefore fundamental for adequate post-treatment CT/MRI interpretation and to guide hepatologists and oncologists towards the best patient management.

### Pancreas

Clinical and radiological manifestations of pancreatic toxicity during systemic ChT and abdominal RT range from asymptomatic increase in blood pancreatic enzymes levels, to acute pancreatitis (AP) or parenchymal atrophy with exocrine insufficiency. In addition, ChT/RT for pancreatic ductal adenocarcinoma (PDAC) may induce changes in peri-pancreatic tissues, particularly at the interface between tumor mass and peri-pancreatic vessels. While asymptomatic increase in serum levels of pancreatic enzymes have been found in up to 30–56% of patients treated with sunitinib and sorafenib, ChT-related AP seems to be much less frequent, although its true incidence is unknown, as the causal relationship between the drug and AP may be difficult to determine [57]. Several drugs can induce AP: all-trans retinoic acids, cytarabine, and L-asparaginase used to treat leukemia, vascular endothelial growth factor receptor (VEGFR) and tyrosine kinase inhibitors, ICIs and drugs like gemcitabine and capecitabine, which are commonly used in PDAC treatment [10, 57]. It has been postulated that capecitabine- and pazopanib-induced hypertriglyceridemia may lead to AP [57]; other factors that can contribute to AP can be a direct toxic effect, an allergic reaction or immune-related events. In general, imaging findings of ChT-associated AP do not differ from their non-drug-induced counterparts: enlargement of the pancreas, decreased parenchymal enhancement, peripancreatic fat stranding, fluid collections, and, in severe forms, intra- and peri-pancreatic necrosis.

**Fig. 18:**
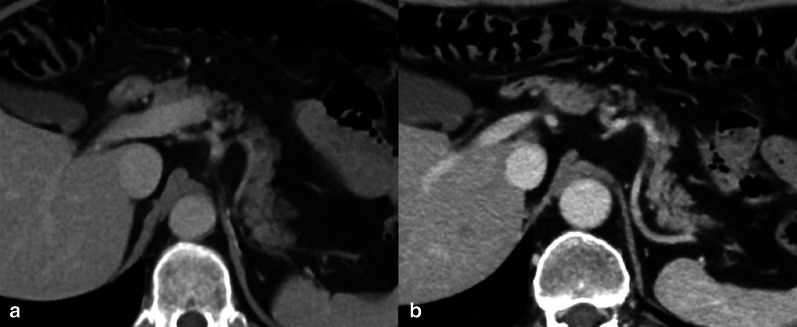
Pancreatic atrophy in a 50-year-old man with metastatic clear cell renal cell carcinoma. Baseline axial CT scan show mild fatty infiltration of the pancreatic parenchyma (**a**). Axial CT scan performed after 2 years of sunitinib treatment (**b**) shows advanced pancreatic atrophy with the decrease in size of the pancreatic body/tail, fatty-fibrous replacement, increased lobular appearance of the gland and decreased attenuation

Pancreatic atrophy is associated with long-term use of sunitinib and sorafenib [10, 57, 58]. Also ICIs can induce irreversible pancreatic atrophy that can lead to exocrine pancreatic insufficiency [59]. Imaging features are comparable to the fibrous-fatty involution commonly seen in elderly: parenchymal thinning with increased peripheral lobulations, change in the parenchymal appearance (hyperechoic at ultrasound, hypodense at CT, and hypointense on T1-weighted MRI), and delayed peak of parenchymal enhancement (Fig. 18).

RT is a known potential cause of bowel damage and chronic pancreatitis. Stereotactic body RT has gained particular interest over recent years, as—compared to conventional RT—it maximizes the therapeutic window by delivering ablative doses to the tumor area, including the peripancreatic vessels, reducing the dose to neighboring organs, such as duodenum, stomach and intestine. Nevertheless, early and late bowel toxicities have been reported also after stereotactic body RT, including stricture, obstruction, ulceration, bleeding, and perforation [60].

**Fig. 19:**
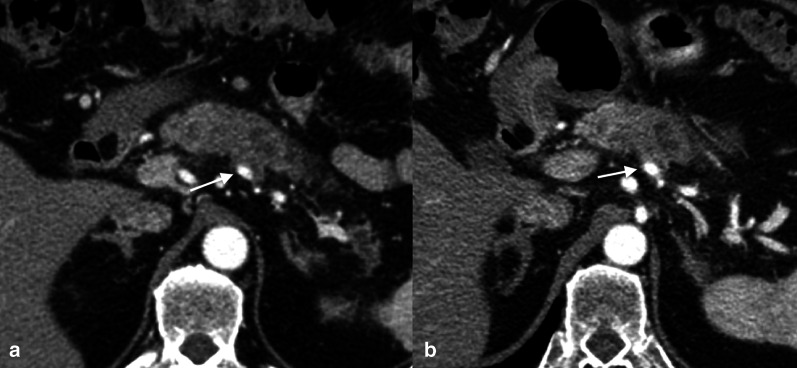
Effects of neoadjuvant ChT/RT in a 63-year-old male with pancreatic body ductal adenocarcinoma. Baseline CT scan (**a**) shows a hypodense lesion consistent with pancreatic ductal adenocarcinoma in the pancreatic body infiltrating the splenic artery (arrow); concurrent infiltration of the celiac trunk and the splenic vein was present. In the CT scan (**b**) performed after neoadjuvant ChT/RT (FOLFIRINOX + stereotactic body RT), the tumor mass was slightly reduced in size, but persistent soft-tissue attenuation was present around the splenic artery (“perivascular cuff”—arrow). The patient underwent surgical exploration with successful distal pancreatectomy and celiac artery resection (Appleby procedure); pathological examination of the resection specimen showed absence of residual disease, negative resection margins, and fibrosis around the splenic artery

One of the most important drawbacks of neoadjuvant ChT-RT for PDAC is that these treatments may induce the development of fibrosis as well as local and regional edema consequent to lymphatic congestion, that result in persistent thickening of the perivascular fat, which may be misinterpreted as persistent vascular invasion (Fig. 19). Indeed, previous studies showed that these “perivascular cuffs” are not correlated to the resection margin status, and that lead to underestimate the radiological evaluation of PDAC resectability after neoadjuvant therapy.

### Gastrointestinal

The gastrointestinal (GI) system is frequently affected by ChT/RT-induced toxicity because of the rapid turnover of enteric epithelium.

Acute esophageal toxicity may occur during or immediately after irradiation of the thorax and usually resolves within 4–6 weeks [61]. Clinically, the most common symptoms include dysphagia, odynophagia and chest pain [61, 62]. Late toxicity may also occur within months to years from thoracic irradiation, with paucisymptomatic course or onset of dysphagia due to fibrotic stenoses [63]. In acute phase, impaired esophageal peristalsis is commonly found at fluoroscopy usually 4–12 weeks after RT, while post-actinic fibrotic strictures with smooth and long stenotic segments with tapered margins usually occur with a median onset of 6 months from irradiation [62]. More rarely, chronic ulcerations, intermittent esophageal spasms, bleeding, perforation and/or fistulation may also occur [61, 63]. At CT, symmetric thickening of the esophageal walls can be appreciated in both early and late toxicity [62]. Along with the esophagus, even stomach and duodenum may be injured after RT. Gastric and duodenal ulcerations may be found, as well as impaired peristalsis of the antro-pyloric region, lumen abnormalities and narrowing [64]. Occasionally, nonspecific thickening of the gastric walls with stranding of the perivisceral fat may be evidenced on CT [64].

In the lower GI tract, the small bowel is more susceptible to RT-induced injuries due to its rapid cellular turnover, with the rectum being the least radio-sensitive part [9]. Acute intestinal toxicity presents with diarrhea, while malabsorption is the main clinical feature in chronic enteropathy [9]. In the first days/weeks after RT, small bowel dilation with submucosal edematous thickening and mucosal enhancement after contrast injection are observed at cross sectional imaging [9, 65]. In chronic phase, the bowel loops are tethered with altered motility. Fibrotic strictures may lead to small bowel obstruction and, sometimes, fistulae can be identified [9, 64, 66]. At fluoroscopy, the small bowel loops are thickened with altered peristalsis and straightening of the mucosal folds, while the large bowel shows a reduced distensibility with strictures and widening of the pre-sacral space. Mucosal ulceration, pseudo-polypoid protrusions, or contour irregularities can also be found [64]. The same findings may be detected by CT and MRI, which are essential to identify fistulae [64].

**Fig. 20:**
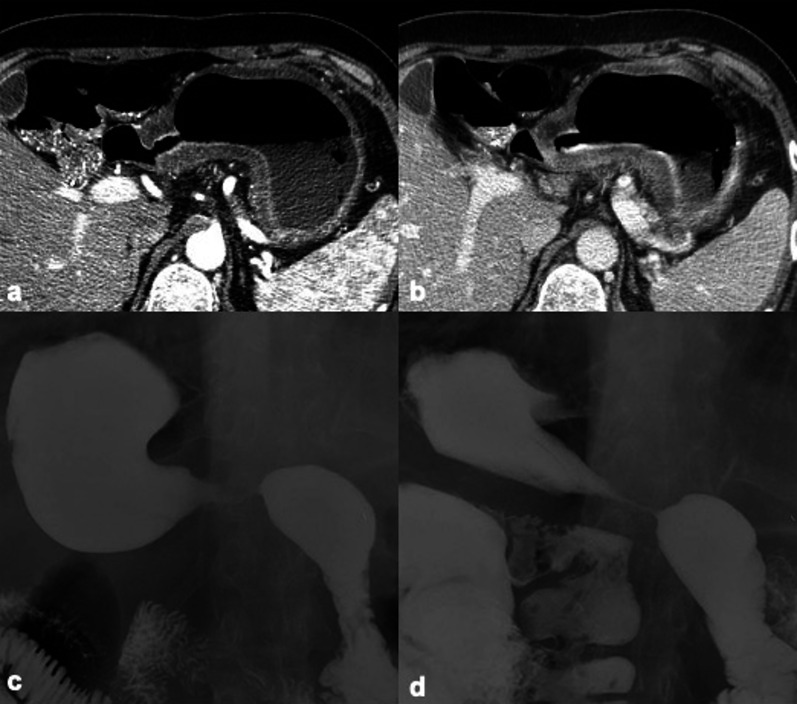
Gastritis due to checkpoint inhibitor therapy (Nivolumab). CT scans in arterial (**a**) and venous (**b**) phase showing linear enhancement of the gastric mucosa and diffuse, marked edematous thickening of the submucosal layer, more evident in the prepiloric area with concentric stenosis of the antral lumen. Moderate perigastric fat stranding is appreciable. The stenotic visceral segment is confirmed at barium fluoroscopy (**c**, **d**), however the passage of the contrast medium at the level of duodenum and small intestine is allowed

GI toxicity is frequently observed also after ChT [67]. Enteritis is often diffuse or prevailing in the distal ileum, while gastritis and duodenitis are less common (Fig. 20). Clinical picture includes abdominal pain, bloating and diarrhea [33]. CT shows the typical “target sign”, characterized by hyper-enhancement of mucosal and serosal layers of the bowel loops with regular thickening and edema of the submucosa. This sign is reliable in differentiating benign conditions from irregular neoplastic thickening of GI walls. Nevertheless, several benign disorders can present this sign and cannot be differentiated on the basis of this imaging feature, including ChT/RT-induced enteritis, ischemic bowel disease, crohn/ulcerative colitis, intramural bowel hemorrhage, and intestinal infections [68]. Bowel loops dilation with air-fluid levels are also common findings, with ileus as a potential complication particularly after treatment with vinca alkaloids [33]. Bleeding and bowel perforation are infrequent toxicities of some anti–VEGFR antagonists such as bevacizumab. In the setting of severe ChT-induced immunosuppression, neutropenic enterocolitis may occur with intramural bacterial invasion and manifestations ranging from mucosal inflammation to transmural wall necrosis. The cecum and the right colon are the most common sites involved in this condition [33]. Further, pneumatosis intestinalis may occur in patients treated with classic ChT agents as well as with Bevacizumab [33]. It is characterized by the presence of gas along the bowel wall with subserosal or submucosal location due to the disruption in mucosal integrity.

### Gynecologic

ChT is used as adjuvant treatment after surgery in advanced ovarian and endometrial cancers, whereas adjuvant RT is used for node positive cervical cancer and endometrial carcinoma extending beyond the myometrium. Primary ChT/RT is used for FIGO stage II-IV cervical cancer [69, 70]. In this scenario, paraphysiological imaging changes of normal gynecologic organs induced by treatment should be distinguished by complications and disease recurrence, with MRI being the most suitable diagnostic modality for this task [69, 71]. About 6 months after pelvic ChT/RT, the uterus reduces in volume and endometrium becomes thin with loss of the distinction between junctional zone and myometrium. Cervical stroma shows low signal intensity on T2-weighted MR images. Also the ovaries reduce in volume and signal with loss of follicular activity [72].

Response to treatment is assessed on the basis of reduction in tumor size and physiological reconstitution of uterine anatomical subdivision and homogeneous low signal intensity of cervical stroma; in addition, DWI may be helpful in distinguishing between residual disease and inflammatory tissue, since the latter does not demonstrate restricted pattern of diffusion [73].

**Fig. 21:**
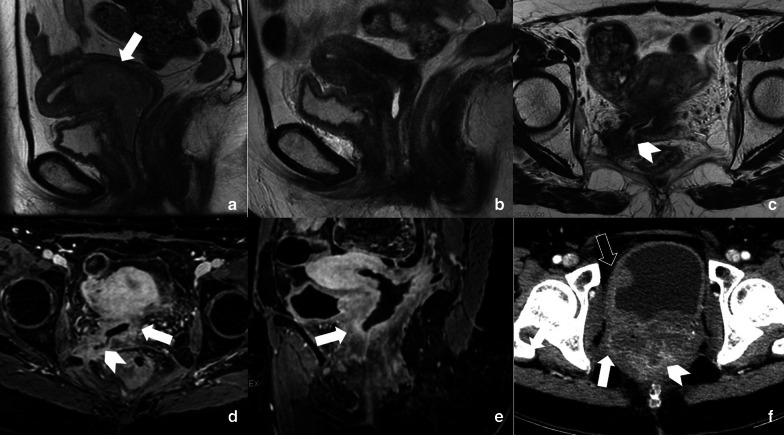
A 52-year-old woman with a FIGO IIa cervical cancer (arrow in **a**) treated with external beam RT and ChT, followed by high dose rate brachytherapy boost in July 2017. In December 2017 (**b**) sagittal T2-weighted image showed an almost normal reconstitution of hypointense cervical stroma. Axial T2-weighted MRI follow-up in April 2018 showed a fistulous tract (arrowhead in **c**) of the right-posterior vaginal wall at the level of fornix, that blinded-ended in correspondence of piriformis muscle. Axial post-contrast fat-suppressed T1-weighted images (**d**, **e**) showed better the fistulous tract (arrowhead in **d**) and irregular enhanced thickening of vaginal wall (arrows in **d**, **e**), extended to cervix, which raised a strong suspicion of disease recurrence. Axial CT image performed on September 2018 showed progressive disease since the pathologic tissue infiltrated pelvic side wall, rectum and bladder (respectively arrow, arrowhead and empty arrow in **f**)

Considering the most frequent complications of RT, cervical stenosis must be taken into account in the acute phase (3–6 months). The resulting obstruction can lead to fluid accumulation in the uterus, causing hematometra with subsequent infection and pyometrium. Fistulas are another fearsome late complication of RT, mostly occurring between bladder and vagina or rectum. Fistulous tracts are well identified on heavily T2-sequences, but contrast medium administration may help to highlight a recurrent disease (Fig. 21) [70].

### Urinary and prostate

ChT/RT effects on urinary system and prostate are relatively common and the diagnosis is usually based on clinical and histological data. Imaging has a well-established role in the diagnostic work-up of tumors of the urinary system [74, 75], but it is not routinely used to reach the diagnosis of ChT/RT-related kidney injury. A common ChT-related damage is acute kidney injury (AKI), affecting around 20% of cancer patients in the first year of disease [76]. Traditional ChT and emerging target therapies can induce kidney microvascular, tubular and/or glomerular acute impairment that may result in interstitial fibrosis, chronic kidney disease (CKD) and dialysis. MRI has been proposed to early detect AKI [77, 78]. Dong et al. tested the ability of arterial spin labeling perfusion technique reporting significantly reduced perfusion in AKI patients both in cortex and medullary [77]. Li et al. [78] proposed blood oxygenation level-dependent functional MRI to identify tissue hypoxia, one of the leading causes of chronic disease development, reporting significant reduction of blood oxygen in renal cortex of CKD patients. However, the reference standard for interstitial fibrosis and CKD diagnosis is currently kidney biopsy, while US is used as initial Imaging method for cortex thickness assessment.

Pelvic RT could induce both acute and late adverse events on prostate and bladder [79, 80]. The main injury of bladder is hemorrhagic cystitis, which is diagnosed on the basis of clinical data and exclusion of other causes, with imaging being used to rule out other causes of hematuria and urinary symptoms [81]. Further, in chronic phase bladder becomes poorly distensible due to fibrosis. Ureters are less affected by pelvic RT, although their involvement may determine stricture and should be considered when dealing with hydronephrosis without obstructing calculi.

**Fig. 22:**
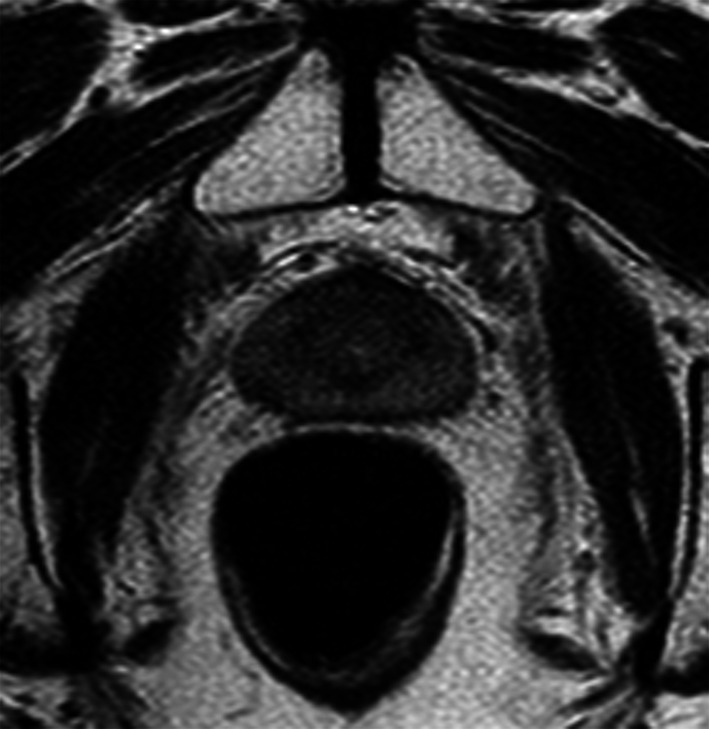
Follow-up MRI scan performed by a 67-year-old man with low-risk prostate cancer 12 months after external beam RT. Axial T2-weighted image shows the loss of zonal differentiation of the prostate

**Fig. 23:**
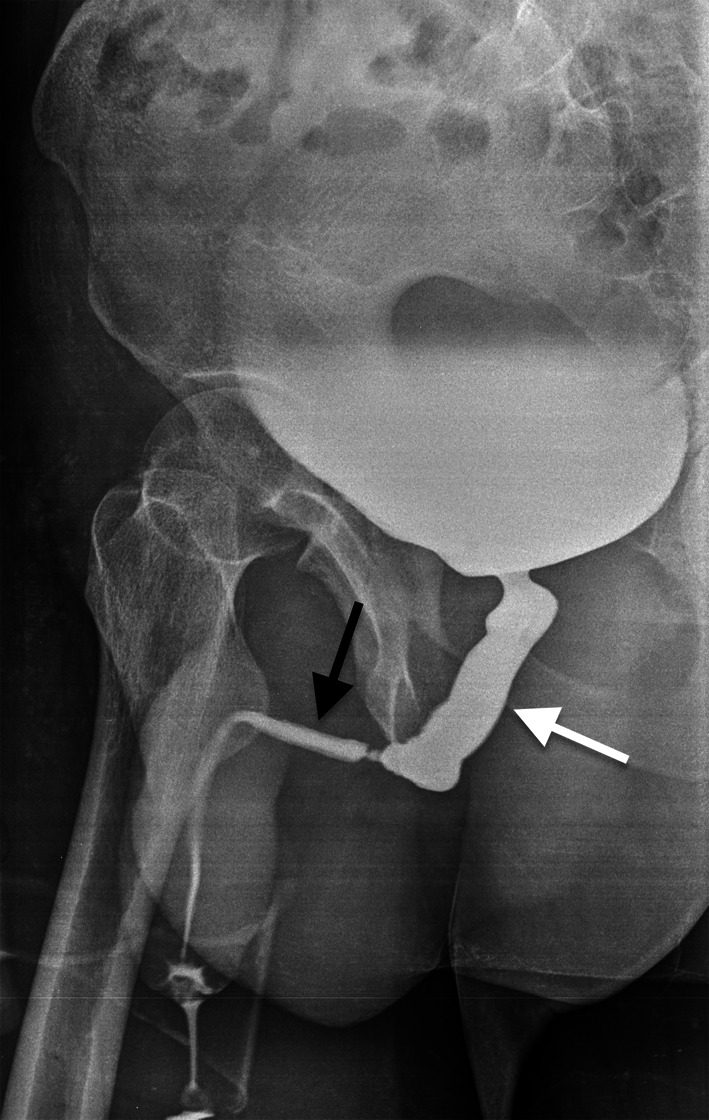
Urinary disorders in a 72-year-old man after pelvic RT due to prostate cancer. Retrograde cystourethrogram, oblique view, shows dilatation of the membranous urethra (white arrow) due to a long stenosis of the bulbar segment (black arrow)

RT is a common therapeutic choice in prostate cancer, it can be performed by external beam RT or brachytherapy [82–85]. RT may induce physiological changes that must be correctly interpreted given that they can hinder tumor detection as well as mimic tumor recurrence. After RT, prostate demonstrates atrophy and diffuse low T2 signal, with loss of normal zonal anatomy (Fig. 22). Moreover, irregularity and stranding of periprostatic fat and atrophy of seminal vesicles can be detected. Therefore, DWI and dynamic contrast enhanced imaging are essential to detect recurrence, showing low ADC value and hypervascularity of recurrence, while high ADC value and slow enhancement in normal fibrotic tissue. After prostatic RT, urethral stricture is a common complication. In particular, until 9% of men after brachytherapy and 13% after external beam RT will develop stenosis [86]. Generally, urethral stricture affects the bulbomembranous urethra (> 90% of cases), while the intraprostatic urethra is less commonly involved even if it receives higher doses of ionizing radiations during RT. In this setting, retrograde cystourethrography and voiding cystourethrography are the most accurate techniques to detect urethral stricture (Fig. 23) [86].

### Spleen and lymph nodes

Systemic ChT and RT targeted to neighboring abdominal tumors may determine changes in splenic appearance that radiologists should take into account. These findings might be both expected effects without clinical impact and complications with non-negligible clinical relevance. Splenomegaly has been reported in association with some ChT protocols. In particular, splenic enlargement can be observed in 50%-87.9% colon cancer patients treated with oxaliplatin-based ChT with 31% median change of spleen size [87]. This finding seems to be a surrogate of oxaliplatin-induced SOS, above mentioned among ChT-induced liver injuries, that can be complicated by thrombocytopenia, which has been confirmed to be higher in colon cancer patients treated by oxaliplatinum and developing splenomegaly after ChT [87]. Thus, this imaging feature encountered in follow-up imaging examinations of oncology patients may be a warning sign in terms of developing SOS. Notably, a previous study has shown that bevacizumab administration in addition to oxaliplatin-based ChT may avoid splenic enlargement, leading the authors to consider the inhibition of splenomegaly as a potential indicator of the protective effect of bevacizumab against SOS induced by oxaliplatin in colon cancer patients [88]. Indeed, patients treated with oxaliplatin only and those treated with oxaliplatin + bevacizumab had 39.1% vs. 2.3% increase of splenic volume and 50% versus 16% incidence of moderate/severe SOS, respectively [88]. If on one hand splenic enlargement may be a side effect of ChT, on the other unintended irradiation of the spleen during RT performed for neighboring malignant tumors may lead to global or segmental splenic atrophy and calcific deposits in splenic parenchyma [64].

Concerning lymph nodes, these are common locations of metastases of solid tumors and lymphoproliferative disorders. Thus, increased size and morphology changes of lymph nodes are always considered as a *wake-up call* when encountered in follow-up imaging examinations performed on oncology patients. Nevertheless, the enlargement of benign lymph nodes may occur in oncology patients subjected to ChT. Hayashi et al. [89] reported the high incidence of early enlargement of benign lymph nodes after ChT/RT in patients with esophageal squamous cell carcinoma, accounting for more than 50% of all enlarged lymph nodes identified on follow-up CT examinations. In most patients (83%), benign lymph nodes enlargements showed no pathologic FDG uptake on PET/CT. Further, these lymph nodes presented decrease or no further changes in size at subsequent follow-up CT scans.

**Fig. 24:**
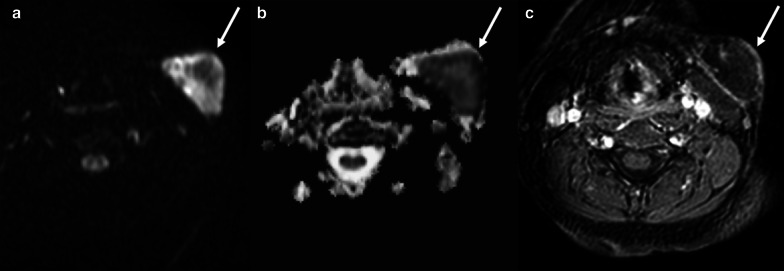
A 62-year-old female patient with pain and swelling in the left side of the neck, fever, and history of larynx cancer treated with RT. Axial high b-value (b = 1000) DWI (**a**), ADC map (**b**), and post-contrast gradient-echo fat-suppressed T1-weighted (**c**) images show an enlarged left laterocervical lymph node (arrows), with very low ADC values (**b**) and no contrast enhancement (**c**) due to the purulent content of suppurative lymphadenitis

Regarding RT of normal or pathologic lymph nodes, a well-known complication is lymphedema related to scarring that interferes with lymphatics functions leading to lymphatic swelling [90, 91]. It starts 2–3 months after initiation of RT and can be highly disabling determining edema and fibrosis of skin/soft tissues and deep structures (e.g. pharyngeal and laryngeal mucosa in HN tumors)[90]. Another possible complication is infection with occurrence of lymph nodal abscess. The diagnostic task of radiologists is to differentiate necrotic neoplastic lymph nodes from abscesses. Indeed, both present fluid content and enhancing peripheral solid tissue on contrast-enhanced CT and MRI. However, DWI can be helpful allowing to distinguish the necrotic core of metastatic lymph nodes (with high ADC values) from the purulent content of suppurative lymphadenitis (with low ADC values) (Fig. 24) [92].

### Bone and soft tissues

**Fig. 25:**
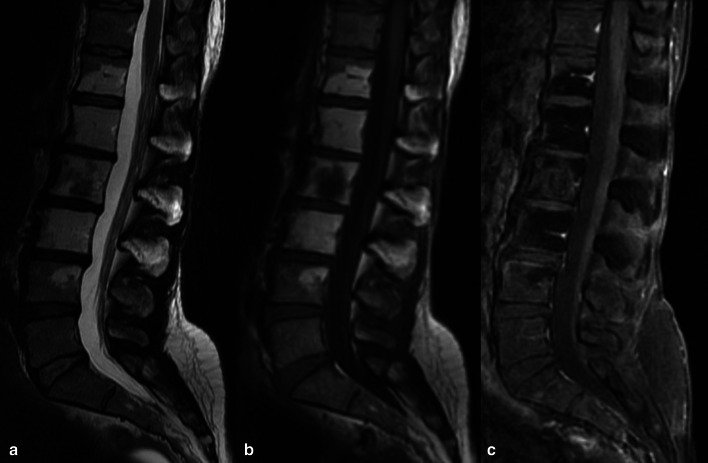
A 46-year-old female patient with history of clear cell renal carcinoma. Sagittal T2-weighted (**a**), T1-weighted (**b**) and contrast-enhanced T1-weighted (**c**) images of the lumbar spine at the level of L2 a bone metastasis previously treated by thermoablation and radiotherapy. Note the bone marrow signal intensity of the vertebral elements in D12-L4 within the irradiation field, due to medullary depletion and fatty replacement

**Fig. 26:**
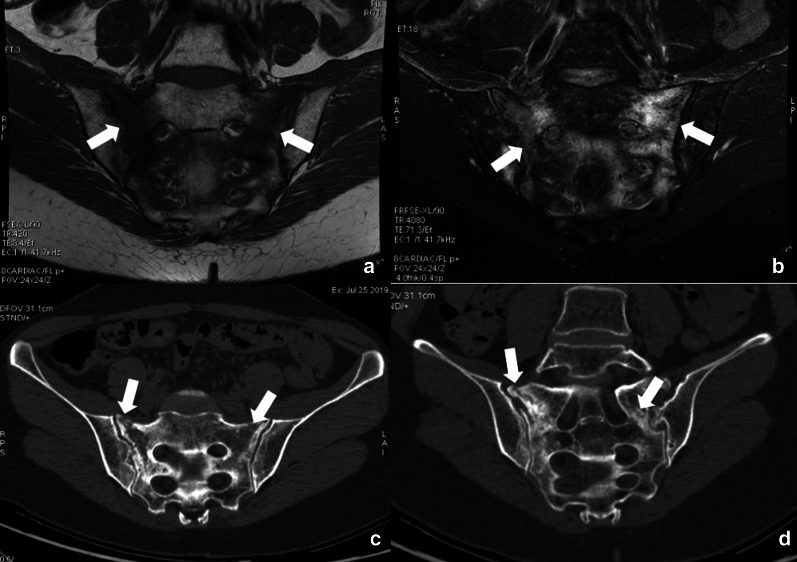
A 54-year-old woman, who underwent RT/ChT for cervical cancer, complained of low back pain 8 months after treatment completion. MRI exam with coronal oblique T1-weighted and fat suppressed T2-weighted images showed extensive bone marrow edema of the sacrum (arrows in **a**, **b**) due to insufficiency fractures clearly identified also by CT images (arrows in **c**, **d**)

**Fig. 27:**
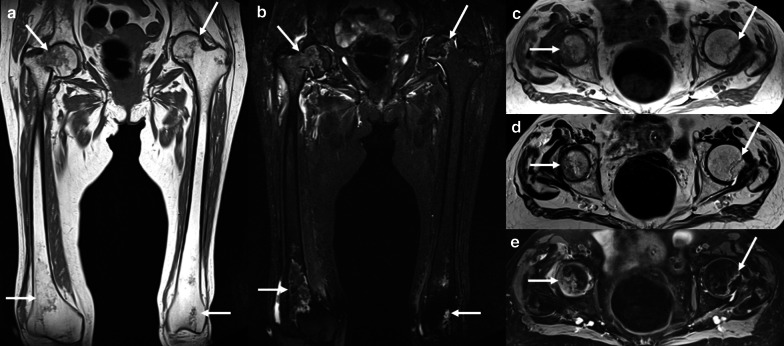
A 68-year-old man with Hodgkin Lymphoma treated with ChT (including high doses of corticosteroids) and atraumatic right hip pain. Coronal T1-weighted (**a**), coronal STIR (**b**), axial T1-weighted (**c**), axial T2-weighted (**d**), and axial proton density-weighted (**e**) images show geographical areas in both femurs with central fat signal, bordered by a serpiginous peripheral line, due to multifocal osteonecrosis (arrows). As a complication of osteonecrosis, a pathologic fracture of the neck of the right femur with bone marrow and perischeletric edema was identified (**a**, **b**). Also note the typical “double line sign” of the osteonecrotic area of the left femur head consisting of inner bright line (granulation tissue) and outer dark line (sclerotic bone) on axial T2-weighted image (**d**)

In the mature skeleton radiations primarily damage the osteoblasts leading to reduced bone matrix production, with unaffected osteoclasts resorption activity, and resulting in progressive osteopenia, coarse trabeculae formation and sclerotic focal areas, which may be radiographically detected after 1 year [93]. Radiations interfere with chondrogenesis in the immature skeleton, with epiphyseal chondrocytes being extremely radiosensitive. On imaging, changes may be seen as metaphyseal sclerosis, widening and fraying of growth plates, that may take up to 6 months to appear. Varus/valgus deformities are reported as pediatric irradiation sequelae, together with limb shortening; the younger is the subject, the greater is the growth impact [93]. Regardless the age, RT causes myeloid depletion due to the radiosensitivity of hematopoietic elements. This results (after 6–8 weeks) in the replacement of hematopoietic elements with adipocytes, with subsequent increase in T1 signal intensity due to fatty conversion (Fig. 25) [93, 94]. Indeed, bone signal depends on marrow cellularity, which is related to several factors including gender, inflammatory conditions, metabolic factors, and marrow activation that can be observed seen in some tumors [95]. Long-term effects of ChT include bone loss with the risk of developing osteoporosis related to traditional ChT agents (e.g. doxorubicin, carboplatin) and/or hormonal therapies. Reduced bone mass with subsequent increased risk of fracture may deserve bone mineral density assessment with dual energy X-ray absorptiometry [96, 97]. The ChT/RT-related weakening of bone segments may lead to insufficiency fractures, which are common sequelae of radiation osteitis, with sacrum and pelvic bones being the most involved sites (Fig. 26). The risk factors associated with insufficiency fractures are advanced age, post-menopausal state, osteoporosis and lower body mass index [70, 98, 99]. Sapienza and colleagues [100] analyzed 21 studies including 3929 patients treated by RT for gynecologic cancers and found that 14% developed insufficiency fractures. Interestingly, 40% of patients with fractures were asymptomatic; therefore post-treatment bone surveillance is warranted to prevent complications [100]. Another complication of both ChT and RT is osteonecrosis. The most common cause of osteonecrosis (10–30% of all cases) is the administration of high doses of corticosteroids, which are indeed included in several ChT regimens [101]. MRI is the imaging of choice for detecting osteonecrosis that presents as geographical areas bordered by a serpiginous T1-hypointense peripheral line, hyperintense on T2-weighted images with the typical “double line sign” consisting of inner bright line (granulation tissue) and outer dark line (sclerotic bone) observed on T2-weighted (Fig. 27) [102]. MRI is also essential to identify complications of osteonecrosis such as articular collapse, osteochondral fragmentations, fractures, and osteoarthritis. Of note, the risk of bone sarcoma secondary to RT is quite low (about 0.005%), with osteosarcoma being more than half of the cases [103]. According to most authors, secondary osteosarcomas have poorer prognosis than primary tumors due to high-grade of tumor, surgical difficulties, and reduced dose of RT that can be used in a previously irradiated field.

RT/ChT can have different effects on soft tissues, particularly on subcutaneous tissues, tendons, and muscles. Imaging findings of tissue changes are often non-specific and a significant overlap can be seen with degenerative and traumatic conditions. After RT, the typical finding is subcutaneous fibrosis of the treated area, with sclerosis and adhesions to underlying tissues. The effects of RT on tendons and ligaments are also characterized by progressive fibrosis with consequent loss of elasticity, shortening, and contracture [104].

Among the effects of drug therapies, it is well known the toxicity on tendons induced by quinolones antibiotics, especially involving the Achilles tendon [105]. Concerning drugs commonly included in ChT regimens, prolonged use of glucocorticoids is another recognized cause of drug-induced tendon degeneration and rupture. Among drug-induced myopathies, several ChT drugs may cause muscle weakness, myopathy, up to rhabdomyolysis in the most severe cases, including ifosfamide, gemcitabine/paclitaxel, cytarabine, doxorubicin, vincristine, mitoxantrone/cyclophosphamide, and cyclophosphamide [106]. Imaging findings are nonspecific, with MRI evidence of muscle edema, bilateral and symmetric muscles enlargement, particularly involving the buttocks and lower limbs.

## Conclusions

ChT/RT-induced changes and complications may present with tricky imaging features that result in challenges in the interpretation of imaging follow-up exams. An accurate interpretation of these findings requires knowledge of therapeutic history, including ChT regimens and RT schemes. Indeed, newer biologic drugs and immunotherapies, as well as more effective and targeted RT, besides inducing tumor changes like pseudoprogression that are changing response criteria in clinical trials, have improved life expectancy of oncology patients thereby increasing the risk of long-term therapy-related side effects. Radiologists are tasked to distinguish expected findings from residual/relapse of tumors and to early identify therapy-related complications, which occasionally may be life-threatening conditions.

## Data Availability

Not applicable.

## Abbreviations

AKI: Acute kidney injury
AP: Acute pancreatitis
ChT: Chemotherapy
CKD: Chronic kidney disease
GI: Gastrointestinal
GGO: Ground-glass opacity
HN: Head and neck
ICIs: Immune checkpoint inhibitors
PDAC: Pancreatic ductal adenocarcinoma
RILI: Radiation-induced lung injury
RT: Radiotherapy
SOS: Sinusoidal obstruction syndrome
VEGFR: Vascular endothelial growth factor receptor
